# Machine learning random forest for predicting oncosomatic variant NGS analysis

**DOI:** 10.1038/s41598-021-01253-y

**Published:** 2021-11-08

**Authors:** Eric Pellegrino, Coralie Jacques, Nathalie Beaufils, Isabelle Nanni, Antoine Carlioz, Philippe Metellus, L’Houcine Ouafik

**Affiliations:** 1 APHM, CHU Nord, Service d’Onco-Biologie, Marseille, France; 2 Departement de Neurochirurgie, Centre Hospitalier Clairval, Marseille, France; 3 Aix Marseille Univ, CNRS, INP, Inst Neurophysiopathol, Marseille, France

**Keywords:** Machine learning, Programming language, DNA sequencing, Next-generation sequencing, Sequence annotation, Bioinformatics, Genetic techniques, Genomic analysis, Sequencing, Software, Cancer genetics, Cancer genomics, Cancer microenvironment, Cancer prevention, Cancer screening, Cancer therapy, CNS cancer, Gastrointestinal cancer, Gynaecological cancer, Head and neck cancer, Lung cancer, Metastasis, Oncogenes, Oral cancer, Paediatric cancer, Sarcoma, Skin cancer, Testicular cancer, Tumour biomarkers, Tumour-suppressor proteins, Urological cancer, Cancer genetics, Cancer genomics, Gene regulation, Genetic markers, Genotype, Mutation, RNA splicing, Sequencing, Biological techniques, Cancer, Computational biology and bioinformatics, Molecular biology, Biomarkers, Molecular medicine, Oncology

## Abstract

Since 2017, we have used IonTorrent NGS platform in our hospital to diagnose and treat cancer. Analyzing variants at each run requires considerable time, and we are still struggling with some variants that appear correct on the metrics at first, but are found to be negative upon further investigation. Can any machine learning algorithm (ML) help us classify NGS variants? This has led us to investigate which ML can fit our NGS data and to develop a tool that can be routinely implemented to help biologists. Currently, one of the greatest challenges in medicine is processing a significant quantity of data. This is particularly true in molecular biology with the advantage of next-generation sequencing (NGS) for profiling and identifying molecular tumors and their treatment. In addition to bioinformatics pipelines, artificial intelligence (AI) can be valuable in helping to analyze mutation variants. Generating sequencing data from patient DNA samples has become easy to perform in clinical trials. However, analyzing the massive quantities of genomic or transcriptomic data and extracting the key biomarkers associated with a clinical response to a specific therapy requires a formidable combination of scientific expertise, biomolecular skills and a panel of bioinformatic and biostatistic tools, in which artificial intelligence is now successful in developing future routine diagnostics. However, cancer genome complexity and technical artifacts make identifying real variants challenging. We present a machine learning method for classifying pathogenic single nucleotide variants (SNVs), single nucleotide polymorphisms (SNPs), multiple nucleotide variants (MNVs), insertions, and deletions detected by NGS from different types of tumor specimens, such as: colorectal, melanoma, lung and glioma cancer. We compared our NGS data to different machine learning algorithms using the k-fold cross-validation method and to neural networks (deep learning) to measure the performance of the different ML algorithms and determine which one is a valid model for confirming NGS variant calls in cancer diagnosis. We trained our machine learning with 70% of our data samples, extracted from our local database (our data structure had 7 parameters: chromosome, position, exon, variant allele frequency, minor allele frequency, coverage and protein description) and validated it with the 30% remaining data. The model offering the best accuracy was chosen and implemented in the NGS analysis routine. Artificial intelligence was developed with the R script language version 3.6.0. We trained our model on 70% of 102,011 variants. Our best error rate (0.22%) was found with random forest machine learning (ntree = 500 and mtry = 4), with an AUC of 0.99. Neural networks achieved some good scores. The final trained model with the neural network achieved an accuracy of 98% and an ROC-AUC of 0.99 with validation data. We tested our RF model to interpret more than 2000 variants from our NGS database: 20 variants were misclassified (error rate < 1%). The errors were nomenclature problems and false positives. After adding false positives to our training database and implementing our RF model routinely, our error rate was always < 0.5%. The RF model shows excellent results for oncosomatic NGS interpretation and can easily be implemented in other molecular biology laboratories. AI is becoming increasingly important in molecular biomedical analysis and can be very helpful in processing medical data. Neural networks show a good capacity in variant classification, and in the future, they may be useful in predicting more complex variants.

## Introduction

Tumor molecular profiles have recently become a major element in diagnosis, classification, and therapeutic management. As a result, the number of molecular analyses has increased considerably. Recently, the next-generation sequencing (NGS), with an oncosomatic gene panel, has become one of the reference techniques used routinely in many laboratories for molecular typing. Data analysis of sequencing requires the use of bioinformatics pipelines to align the sequencing results on the human genome reference (hg19); and to filter mapped reads against the reference genome to perform variant analysis, including variant calling and predicting the effects produced by found variants on genes. After these steps, biologists can begin the interpretation for each patient; they have to check a list of quality parameters to determine the mutation pathogenicity, by establishing the influence these mutations have on the protein. For example, if it is a synonymous variant (p.(=)), it will probably have a low influence on the gene because the amino acid does not change (Supplementary Fig. S8). However, if it is a large deletion, a large effect on gene function is assumed. Artificial intelligence (AI) can be beneficial for all interpretation processes. AI is defined as the set of theories and techniques used to produce machines capable of simulating human intelligence. AI is taking an increasingly important role in daily life, especially in the medical field with the development of biotechnologies. Many studies have been published and show extraordinary results in the healthcare domain, such as radiomic and digital pathology^1^. Machine learning, neural networks and deep learning are part of artificial intelligence. These approaches result in computers being able to make intelligent decisions. Neural networks make up the backbone of deep learning algorithms. We identify feedforward neural networks known as multilayer perceptrons (MLPs) where all arrows go in the same direction (from layer i to layer i+1) of the output and recurrent neural networks (RNNs) which might have a loop and tend to be much harder to train. MLP is characterized by several layers of input nodes connected as a directed graph between the input and the output layers. They use backpropagation to train the network and are widely used for solving problems that require supervised learning as well as research into computational neuroscience and parallel distributed processing. Deep Learning (or a deep neural network) is a network with a certain level of complexity that has more than two layers (or at least one hidden layer between the input and output). It uses sophisticated mathematical modeling to process data. The key to these sophisticated neural networks is dealing with unlabeled or unstructured data. This approach is particularly suitable for complex tasks, when all aspects of the data to be treated cannot be categorized upstream. The deep learning system identifies the discriminating characteristics of the data without the need for prior categorization. In each layer, it searches for new specific criteria of the object, which is used as a basis to determine the classification retained at the end of the process. The system does not need to be trained by a developer; it assesses the need to modify the classification or create new categories based on the new data. While machine learning works from a controllable database, deep learning needs a much larger quantity of data. The system must have many more entries to achieve reliable results. Machine learning is the oldest and simplest technology. It is based on an algorithm that adapts the system based on human feedback. Implementing of this technology implies the existence of organized data. The system is then fed with structured and categorized data, allowing it to understand how to classify new similar data. Based on this classification, the system then performs the programmed actions. In AI, machine learning (ML) is defined by algorithms with the ability to learn without being explicitly programmed. Random forest (RF) is an ML algorithm that is defined by an ensemble of learning methods for classification, regression and other tasks that operate by constructing a multitude of decision trees at the training phase and outputting the classes (classification problem) or mean prediction (regression problem) of the individual trees. RF corrects decision trees to prevent any overfitting during the training phase^2^. The algorithm builds each of its decision tree, training them all with a subset of the problem data. It randomly chooses N records from the data and learns on multiple trees that are randomly built and trained according to the bagging principle. This ensures that all the decision trees have a different experience of the problem. Once all the decision trees have been trained, RF makes its decisions in accordance with the classification or regression problem to be solved by voting on all its decision trees. The RF randomness is found in the sampling on which the trees are built and in the variable selection on which the segmentation is carried out. That is why RF is a black box in many cases. If a forest of 500, 1000, or even 10,000, the mechanism of each tree is usually not of interest. In our study, we are interested in using machine learning and neural networks (MLPs) to interpret NGS oncosomatic results. We focus on the random forest ML algorithm, which offers the best accuracy. Additionally, we attempt to determine whether any neural network architecture can achieve a good somatic classifier. We validate the RF and ANN (artificial neural network) model by comparing the RF and MLP results to biologist decisions.

## Materials and methods

### Methodology and model selection NGS analysis

Our dataset is composed of a cohort of 102,011 SNVs, SNPs and indel (insertion deletion) variants from our 27 gene panel sequenced for colorectal cancer, melanoma, lung cancer and 18 genes sequenced for glioma cancer. It represents a total of 7301 samples. Each SNV, SNP and indel were manually inspected and labeled as either pathogenic (51,008 variants) or benign (51,003 variants) as part of the clinical laboratory workflow. Our panel Oncomine Solid Tumor and Oncomine Solid Tumor + (OST/OST+) contains hotspots of target regions of 27 genes of interest in oncogenesis: KRAS, EGFR, BRAF, PIK3CA, AKT1, PTEN, NRAS, HRAS, STK11, MAP2K1, ALK, DDR2, CTNNB1, MET, TP53, SMAD4, FBXW7, FGFR1, FGFR2, FGFR3, NOTCH1, ERBB4, KIT, PDGFRa, POLE, RET, ROS. Our in-house glioma panel was made to define glioma grades and to target the following genes by sequencing full sequences and hotspots of AKT1, ATRX, BRAF, CDKN2A, CIC, EGFR, FGFR1, H3F3A, HIST1H3B, IDH1, IDH2, NOTCH1, PIK3CA, PIK3R1, PTEN, PTPN11, TERT and TP53.

For all clinical samples, after tumoral genomic DNA extraction by the Maxwell RSC DNA FFPE kit or Maxwell RSC Cell DNA (Promega, France), we performed sequencing with Ion Torrent S5XL (Thermo Fisher, France) with a sensitivity of 5% and minimum coverage of 500X. Then, sequencing data were analyzed through 2 pipelines. The first pipeline was developed by ThermoFisher on the IonTorrent Suite + Ion Reporter. IonTorrent Suite generates FASTQ data and ensures BAM (binary alignment mapping) alignment with the hg19 reference genome by using the TMAP (Torrent Mapping Alignment Program). Ion Reporter makes variant caller and variant annotations. The second pipeline was developed in our laboratory and runs open source software such as BWA-MEM for alignment, SAMtools for mpileup, VarScan2 as variant caller and VEP Ensemble for annotations. All data are stored in our local MySQL database.

Biologists manually validated variants if the following statements were true:

- Variant allele frequency $\ge 5\%$
- Minor allele frequency is absent
- Variant reads $$\ge 500$$
- Amino acid change is different from silent ($\ne p.(=)$) or ($\ne p.?$ for a noncoding region) in cases where the variant’s location is in the exonic region. This statement is not applied, for example, to MET mutations in lung cancer which are in the splice site region and coded as *p*.?. This is also the case for the glioma panel where the TERT mutation hotspot is in the promoter region.
- Variants are present in several oncosomatic databases, such as COSMIC, VarSome, and TP53 IARC, and are known to have pathogenic or uncertain significance.
- Alignment sequences are clear and show no strand bias in the region where the variant is located. This is performed due to the Integrative Genomics Viewer (IGV), which allows us to eliminate false positives.
- In the case of hotspot mutation variants, if it has $reads\ge 500$ and VAF above or below 5%, the variant is validated by a biologist.
- We verify the presence of these pathogenic variants in our second in-house pipeline to validate them.

With these requirements, biologists determine whether a variant can be validated or rejected as a function of the patient’s pathology.

Parameter of ThermoFisher Ion Reporter and Pipeline 2: See Supplementary data.

### Construction of the training dataset and feature selection

Before starting dataset construction, we need to calculate entropy and information gain. Calculating information and entropy is a useful tool in machine learning and is used as the basis for techniques such as feature selection, building decision trees, and, more generally, fitting classification models. Entropy is defined by this equation:

1 $$\begin{aligned} {H_{p}}=H\left( p_{1}\ldots p_{n}\right) =\sum _{i=1}^{n}p_{i}log_{2}p_{i} \end{aligned}$$

where $p_{i}$ is the probability of value *i* and *n* is the number of possible values.

Entropy is the measurement of the impurity or randomness in the data points, and it is calculated between 0 and 1^16^. Entropy is the lowest (no disorder = 0) at extremes (both end = 1) and maximum (high disorder $=\frac{1}{2}$) in the middle. The concept of entropy plays an important role in calculating information gain (IG)^17^. It is applied to quantify which feature provides maximal information about the classification based on the notion of entropy^17^, so it answers this question by measuring how much “information” a feature provides us about the class^18^. The information gain is the product of class probabilities with logarithm base 2 of that class probability.

2 $$\begin{aligned} IG=H_{p}-\sum _{i=1}^{n}p_{ci}H_{ci} \end{aligned}$$

where $H_{p}$ is the entropy of the parent node, $p_{ci}$ is the probability of an observation being in child *i*, and $H_{ci}$ is the entropy of child segment *i*.

#### Construction of the training dataset

Data were extracted from the local MySQL database, and we cleaned and unified the data to make them reliable for any analytic or machine learning process. Some of the recorded data did not follow standard schema or break integrity constraints. Much of the work involved cleaning datasets, such as removing errors and anomalies or replacing observed values with true values, for integration in the training phase. Biologists review variants at each NGS run and determine whether a variant can be validated. Therefore, each variant is flagged YES or NO (in our dataset: $validationVariant=y=\left\{ YES=1;NO=0\right\}$). Yes denotes the variant is considered pathogenic for the pathology and NO denotes benign.

To perform our analysis, we worked on a Linux Centos 7 workstation with 16 GB Memory and an Intel Xeon E5-2603 v3 1.60 GHz CPU (12 cores).

We defined the following data structure for machine learning with 7 parameters:

1. Chromosome (Chr) ${\Rightarrow }$ Chromosome number (integer)
2. Position (POS) ${\Rightarrow }$ Chromosome position (big integer)
3. Exon ${\Rightarrow }$ Integer. Exon is the exon number in each gene (position of exon in a transcript, starting with one for the first exon). Zero is for a not coding region (intronic).
4. Variant allele frequency (VAF) ${\Rightarrow }$ Float
5. Minor allele frequency (MAF) ${\Rightarrow }$ defined as boolean 1 or 0
6. Coverage ${\Rightarrow }$ read depth (big integer)
7. Protein description (protdesc) ${\Rightarrow }$ Amino acid change (boolean) {0: silent, 1: protein change}

A total of 51,008 oncosomatic variants were labeled pathogenic from colorectal, lung, melanoma and glioma cancer. A total of 51,003 variants were considered SNPs or did not have enough requirements to be retained by the biologist.

We formalized the somatic mutation prediction problem as a classification problem. Each candidate mutation site was represented by a feature vector *x* = {chromosome, position, exon, VAF, MAF, coverage, protein description} with 7 inputs described above:

3 $$\begin{aligned} {x=x_{i=1,7}=\left\{ x_{1},\ldots x_{7}\right\} } \end{aligned}$$

The goal was to predict variable $y=${potential pathogenic variant}$=\left\{ \frac{0=FALSE(NO)}{1=TRUE(YES)}\right\}$ with the 7 input features $x_{i}$ (equation 4). If variable *y* was equal to zero, the variant was benign, and if the variable *y* was equal to one, the variant was considered pathogenic.

4 $$\begin{aligned} x_{i}=\left\{ x_{1},x_{2},x_{3},x_{4},x_{5},x_{6},x_{7}\right\} \rightarrow y\overset{?}{=}\left\{ \begin{array}{c} 1=\text {YES (pathogenic)}\\ 0=\text {NO (benign)} \end{array}\right\} \end{aligned}$$

The entropy of our dataset $H=1$, as expected, was different from zero. The entropy for the YES and NO class is zero. This indicates that we acquired a complete knowledge of the contents of this set. If we were asked to draw one observation at random and predict the variant, we knew we would draw a pathogenic or a benign variant.

The information gain *IG* for features contained in our dataset was as follows: chromosome (Chr) 0.06, position (POS) 0.06, exon 0.01, variant allele frequency (VAF) 0.25, minor allele frequency (MAF) 0.36, coverage 0.01 and amino acid change (protdesc) 0.45.

We captured some information from each feature (variable), and amino acid change (protdesc) provided the greatest information gain.

#### Model selection

We compared different predictive models according to our NGS data to choose the best model. First, we created a set of common training controller objects with the same train/test folds and model evaluation metrics (accuracy) that were reused. This was important to guarantee a fair comparison between the different models. We needed to know which to apply to our data, and for that, we used statistical methods to estimate the accuracy of the models that we created on unseen data. We also wanted a more specific estimate of the accuracy of the best model on unseen data by evaluating it on actual unseen data. For that, we held back some data that the algorithms did not see, and we used these data to obtain a second and independent idea of how accurate the best model was. The number of instances (rows) that belonged to each class provided the class distribution of our data. To create and estimate the accuracy of some models of the unseen data, we began by setting-up the test harness to use 10-fold cross-validation^3^. Then we built 5 different models to predict a potential pathogenic variant (*y*) from variant features to finally select the best model. This split our dataset into 10 parts, trained in 9 and tested on 1 and released for all combinations of train-test splits^19^.

We repeated the process 3 times (repeated k-fold) for each algorithm with different splits of the data in 10 groups, to obtain a more accurate estimate^19^. This method is one solution to reduce the noise in an estimated model performance by increasing the k-value. Repeated k-fold cross-validation has the benefit of reducing the bias in the estimated model performance. In our case, k = 10 and repeated = 3, so 30 different models were adjusted and evaluated. We used the accuracy metric to evaluate the models. This is a ratio of the number of correctly predicted instances divided by the total number of instances in the dataset multiplied by 100 to obtain a percentage^19^. We tested 5 ML algorithms mixing linear, nonlinear and complex nonlinear methods (linear discriminant analysis (LDA) $\Rightarrow$ linear, classification and regression trees (CART) $\Rightarrow$ nonlinear, k-nearest neigbords (kNN) $\Rightarrow$ nonlinear, support vector machines (SVM) $\Rightarrow$ complex nonlinear, random forest (RF) $\Rightarrow$ complex nonlinear). At each algorithm run, we reset the random number seed to ensure that the evaluation of each algorithm was performed with the same data splits.

## Results

**Figure 1:**
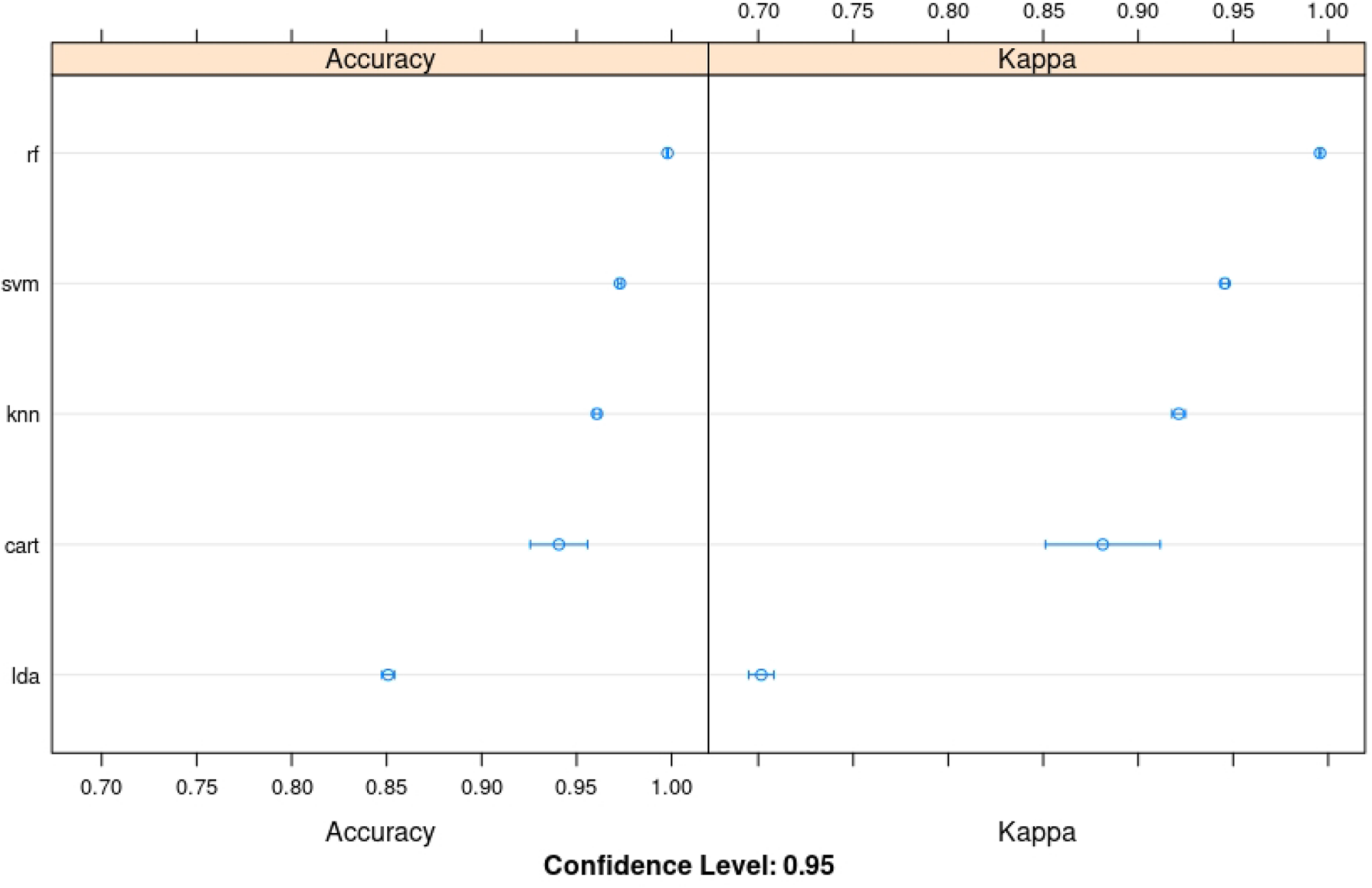
ML algorithms comparison: linear discriminant analysis (LDA) $\Rightarrow$ linear, classification and regression trees (CART) $\Rightarrow$ nonlinear, knNearest neigbords (kNN) $\Rightarrow$ nonlinear, support vector machines (SVM) $\Rightarrow$ complex nonlinear, random forest (RF) $\Rightarrow$ complex nonlinear. Number of resamples: 30.

Model selection with 30-fold cross-validation was performed with 70% of the dataset (train set), and 30% of the remaining data were used for validation. The data were randomly split into two mutually exclusive subsets: training and validation sets. The results of 30-fold cross-validation on the 5 models are as reported in (Table 1 and Fig. 1). According to the results presented in Fig. 1 and Table 1, we can see the accuracy of each classifier and metric such as kappa (see Table 3 for kappa details). The plot of the model evaluation results allows us to compare the spread and the mean accuracy of each model. The accuracy measures for each algorithm are populated because each model was evaluated 30 times (10-fold cross-validation with repeated k-fold cross-validation). Based on the obtained data, it is clear that the random forest is the most accurate model with our NGS dataset.

**Table 1:** Accuracy of ML algorithms after 30-fold cross-validation. The minimum (min), 1st quartile (1st Qu.), median, mean, 3rd quartile (3rd Qu.), maximum (max) are calculated for each machine learning algorithm tested on the oncosomatic variant dataset. 1st Qu. is the median (the middle) of the lower half of the data, approximately 25% of the numbers in the dataset lie below 1st Qu and 75% lie above the 1st Qu. The 3rd Qu. is the median (the middle) of the upper half of the data, about 75% of the numbers in the dataset lie below 3rd Qu. and approximately 25% lie above the 3rd Qu.

Accuracy							
	Min.	1st Qu.	Median	Mean	3rd Qu.	Max	NA’s
lda	0.8431757	0.8468371	0.8521627	0.8508151	0.8545082	0.8562500	0
cart	0.9169219	0.9226504	0.9408207	0.9406823	0.9603283	0.9638525	0
knn	0.9578483	0.9581253	0.9607279	0.9606788	0.9630855	0.9637344	0
svm	0.9693665	0.97237720	0.9731021	0.9727975	0.9734996	0.9745129	0
rf	0.9974268	0.9974577	0.9977943	0.9978557	0.9980394	0.9986525	0

Random forest results across tuning parameters after 30-fold cross-validation can be summarized:

- mtry = 2 Accuracy = 0.9973 and kappa = 0.9947
- mtry = 4 Accuracy = 0.9978 and kappa = 0.9951
- mtry = 7 Accuracy = 0.9975 and kappa = 0.9951

The kappa coefficient obtained with mtry = 4 is 0.9957113 (Table 2), which is above 0.90. According to the kappa Cohen table (Table 3), there is almost perfect agreement. The kappa coefficient is frequently used in statistics to test interrater reliability^20^. The importance of rater reliability lies in the fact that it represents the extent to which the data collected in the study are correct representations of the variables measured^20^. Measurement of the extent to which data collectors (raters) assign the same score to the same variable is called interrater reliability^20^.

**Table 2:** Kappa metric results after 30-fold cross-validation. It compares the accuracy of the models to the accuracy of a random system. This statistic is a measure that can handle both multiclass and imbalanced class problems.

Kappa results							
	Min.	1st Qu.	Median	Mean	3rd Qu.	Max	NA’s
lda	0.6863514	0.6936705	0.7043258	0.7016283	0.7090152	0.7125000	0
cart	0.8338433	0.8453000	0.8816417	0.8813644	0.9206570	0.9277045	0
knn	0.9156957	0.9162500	0.9214557	0.9213574	0.9261705	0.9274688	0
svm	0.9387329	0.9447439	0.9462042	0.9455949	0.9469992	0.9490259	0
rf	0.9948536	0.9949153	0.9955885	0.9957113	0.9960788	0.9973043	0

**Table 3:** Interpretation of Cohen’s kappa values.

Value of kappa	Level of agreement	% of data that are reliable
0–0.20	None	0–4
0.21–0.39	Minimal	4–15
0.40–0.59	Weak	15–35
0.80–0.90	Strong	64–81
Above 0.90	Almost perfect	82–100

After we determined which model to use with our data, it was time to tune the model and test on the validation dataset to make data predictions on future unseen NGS datasets.

### Random forest algorithm training, tuning, testing and predicting

**Figure 2:**
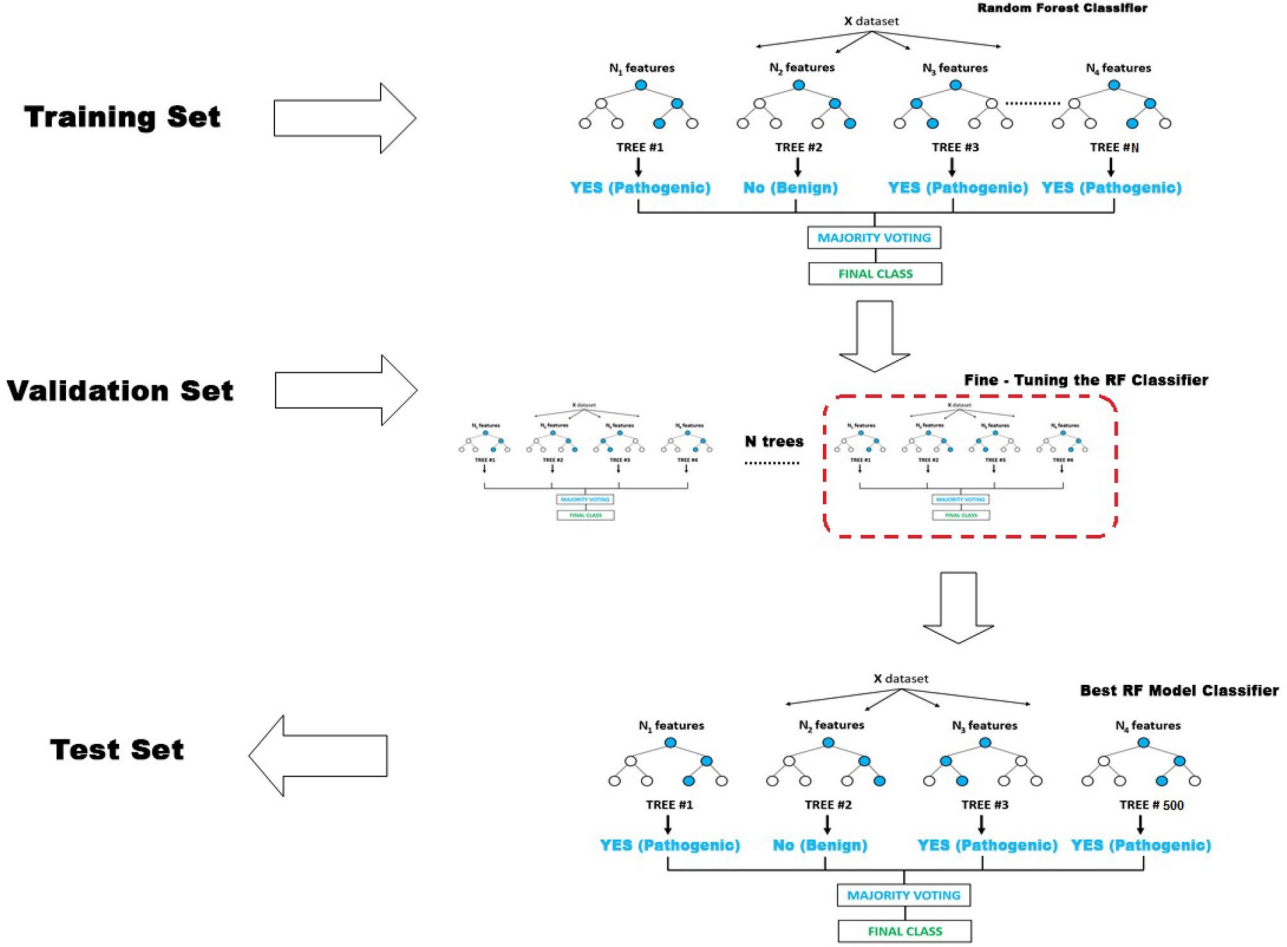
Computational classifier development. The model was trained using the training set. Different sets of parameters were evaluated to identify the best-performing model using a validation set before using the test set in routine NGS.

Random forest produces many small classification trees on a random fraction of the data, and then a voting mechanism occurs^4^ (Fig. 2). From this vote, the order and the importance of the explanatory variables are deduced. We used all the decision trees produced to make the prediction, with a majority vote (for classification, predicted variable of factor type), or an average (for regression, predicted variable of numeric type). The decision of the majority then prevailed. Each tree in the forest was built on a fraction (“in bag”) of the data (the fraction that was used for training the algorithm). Therefore, for each individual of the remaining fraction (“out of bag”), the tree predicted a class. The goal was to obtain the smallest possible out-of-bag (OOB) estimate error. The OOB error is a measure of the random forest prediction error and evaluates its performance. We analyzed the OBB error with default parameters, and then we tuned the model with input parameters such as ntree (number of trees), and mtry (number of variables considered for each tree node) to fit the model accuracy. Tuning hyperparameters (ntree, mtry) to achieve two goals: increase the predictive power of the model and make it faster. Mtry refers to the number of variables available for splitting at each tree node. For the classification model, the default is the square root of predictor variables (rounded down); in our case, mtry = 3 was the default. The TuneRF package found the optimal mtry to obtain the smallest OOB. When satisfied with the results classification of the training dataset, we applied the algorithm on new unseen data to test RF prediction. Our results were presented on a confusion matrix (also known as an error matrix): a table presenting the observed data predicted by the RF algorithm demonstrates the performance of the RF algorithm to predict the variable y.

Each row of the matrix represents the instances in a predicted class, while each column represents the instances in an actual class. In abstract terms, the confusion matrix is shown below:

$$\begin{aligned} \left( \begin{array}{ccc} &{} \text {{Predicted NO}} &{} \text {{Predicted YES}}\\ \text {{Actual NO class}} &{} \text {{TN}} &{} FP\\ \text {{Actual YES class}} &{} FN &{} \text {{TP}} \end{array}\right) \end{aligned}$$

where TP = true positive, FP = false positive, FN = false negative, and TN = true negative.

A true positive (TP) is a result where the model correctly predicts the positive class. Similarly, a true negative (TN) is a result where the model correctly predicts the negative class.

A false positive (FP) is a result where the model incorrectly predicts the positive class. A false negative (FN) is a result where the model incorrectly predicts the negative class.

We trained our ML model on 70% of 102,011 variants that is the total of 3 years of NGS analysis in our platform (2017–2019). Our confusion matrix reported 71,249 well-classified variants and 158 poorly classified variants.

$$\begin{aligned} {\text {Confusion matrix on our dataset:}} \left( \begin{array}{ccc} &{} NO &{} YES\\ NO &{} 35{,}550 &{} 158\\ YES &{} 0 &{} 35{,}699 \end{array}\right) \end{aligned}$$

Our best OOB was a 0.22% error rate and was stabilized with ntree = 500 and mtry = 4 (Supplementary Figs. S1, S2). Our strategy to tune our model was to choose the number of trees keeping the default value of mtry and to test several values by evaluating them. Plotting the model helped us choose the best ntree: before 500 trees, all OOB values oscillated excessively, and after 500, they stabilized. Using more than 500 trees, was more machine time consuming, which is why we fixed the value of ntree = 500 (Supplementary Fig. S1). The data demonstrated that the best OOB was clearly improved with mtry = 4 (Supplementary Fig. S2).

To assess the performance of the model, accuracy, recall, and precision were calculated to measure the performance of the classification model. Accuracy is one metric for evaluating classification models. Informally, it is the fraction of predictions that helped us to determine the correct model. Accuracy is defined as the following equation:

$$\begin{aligned} Accuracy=\frac{TP+TN}{TP+TN+FP+FN} \end{aligned}$$

The model achieved 99.77%. This means that our oncosomatic classifier performs well in identifying malignancies of mutation variants. Accuracy alone is not enough when we are working with a class-balanced dataset, such as this one, where there is not a significant disparity between the number of positive and negative labels. We need to evaluate other metrics, such as precision and recall. Precision is defined as follows:

$$\begin{aligned} Precision=\frac{TP}{TP+FP} \end{aligned}$$

Precision attempts to determine the proportion of positive identifications that were actually correct. In our dataset, $\frac{35{,}699}{35{,}699+158}=0.99$. Our model achieved a precision of 0.99, in other words, when it predicted that a variant was pathogenic, it was correct 99% of the time.

Recall (also known as sensitivity^14^) attempts to determine the proportion of actual positives identified correctly. Mathematically, recall is defined as follows:

$$\begin{aligned} Recall=\frac{TP}{TP+FN} \end{aligned}$$

When FN = 0, and $Recall=\frac{TP}{TP}=1$, our model achieved a recall of 1, which means: that it correctly identified 100% of all pathogenic tumors.

Specificity measures the proportion of true negatives. It is the proportion of samples that truly do not have the condition (unaffected) that are correctly identified as not having the condition, and it is defined as follows:

$$\begin{aligned} Specificity=\frac{TN}{TP+FP} \end{aligned}$$

Specificity = 0.99, means that when a variant is not pathogenic (benign), it is correct 99% of the time. In other words, specificity is the opposite of precision.

### Classification: ROC curve and AUC

An ROC curve (receiver operating characteristic curve) is a graph showing the performance of a classification model at all classification thresholds. This curve plots two parameters:

- True positive rate
- False positive rate

The true positive rate (TPR) is a synonym for recall and is therefore defined as follows:

$$\begin{aligned} TPR=\frac{TP}{TP+FN} \end{aligned}$$

The false positive rate (FPR) is defined as follows:

$$\begin{aligned} FPR=\frac{FP}{FP+TN} \end{aligned}$$

An ROC curve plots TPR versus FPR at different classification thresholds. Lowering the classification threshold classifies more items as positive, thus increasing both false positives and true positives^21^. AUC stands for “area under the ROC curve.” The AUC measures the entire two-dimensional area underneath the entire ROC curve from (0,0) to (1,1). AUC provides an aggregate measure of performance across all possible classification thresholds^21^. One interpretation of AUC is as the probability that the model ranks a random positive example more highly than a random negative example.

**Figure 3:**
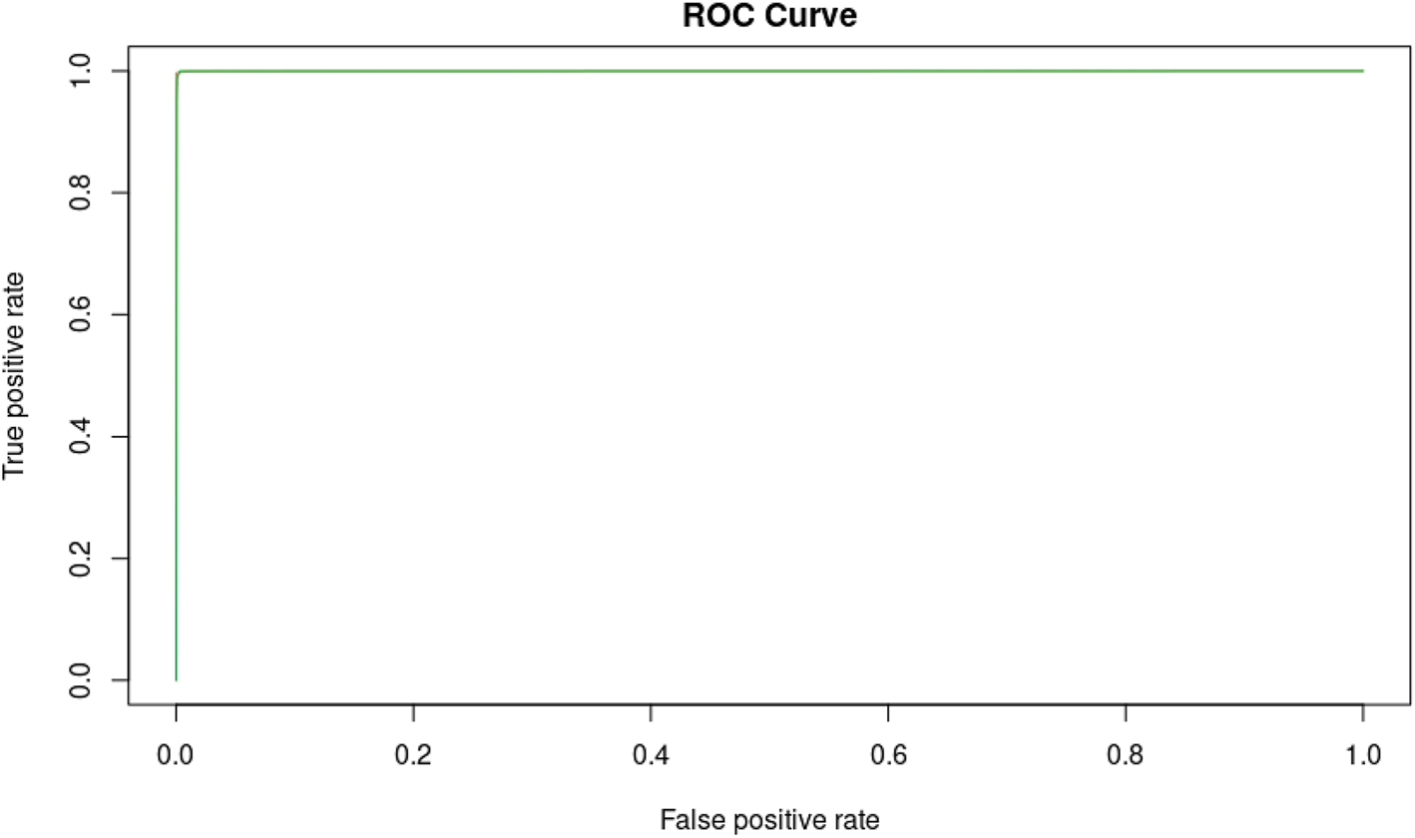
ROC curve for the validation set. Thirty cross-validations were assessed using 7 functional features: chromosome (Chr), position (POS), exon, variant allele frequency (Freq), minor allele frequency (MAF), coverage and amino acid change (protdesc) for the model machine learning random forest (MLRF) for oncosomatic variants. Model performance was evaluated using the receiver operating characteristic (ROC) curve. The area under the curve is denoted AUC. AUC obtained was 0.99. A large area under the curve was observed. The model could then determine whether a variant was benign or pathogenic with a minimal error rate.

If we look at the ROC for our model (Fig. 3), we can see that this is the best possible ROC curve, as it ranks all positives above all negatives. It has an AUC of 0.99 for classes YES and NO labels.

We need now to evaluate the optimal model classifier on the independent validation dataset, which represents the 30% remaining. It was composed of 30,603 SNV variants (15,215 were SNPs and 15,389 were pathogenic oncosomatic variants). Applying the model, gives us:

$$\begin{aligned} \left( \begin{array}{ccc} &{} NO &{} YES\\ NO &{} 15{,}215 &{} 0\\ YES &{} 80 &{} 15{,}309 \end{array}\right) \end{aligned}$$

Of 15,215 TN SNP, 100% were predicted real. A total of 15,309 out of 15,389 (99.5%) were labeled pathogenic. More importantly, none of the TN SNPs were misclassified as pathogenic, while only 0.52% were labeled benign and required further manual investigation to correct them. These 80 are misclassified and 15,309 are correctly classified as pathogenic. This would not have an impact on clinical outcome, as the biologist manually inspected all variants, and the proportions of 0.5% error were very few in comparison with the efficiency of the classifier in predicting the correct class. We created a model that can classify somatic variants with high precision, recall (sensitivity) = 1, specificity = 0.99 and accuracy = 0.997. A high value of accuracy observed on the validation dataset can overfit, but this was not the case. Overfitting is when the model fits well to the training dataset but fails to fit the validation dataset. We observed a good prediction score and no large gap between the training dataset and validation dataset predictions. Moreover, to avoid overfitting, we optimized a tuning parameter that governed the number of features that were randomly chosen to grow each tree from the bootstrapped data. This was done via k-fold cross-validation by choosing tuning parameters (mtry, ntree) that minimized test sample prediction error. The most widely used technique to avoid overfitting is cross-validation^22^.

### Feature importance

To determine the importance of the features and their contribution in our optimal model, we used the mean decrease Gini index. The higher this indicator, the more important the variable is in the model (it measures the decrease in the Gini index if we no longer include this feature in the model).

There is indeed a correlation between these 3 oncosomatic parameters, because a variant comprising an MAF and a silent amino acid change with an allelic frequency close to 90–100% has a high chance of being an SNP (Supplementary Fig. S9). A correlation does exist between amino acid change (protdesc), MAF and variant allele frequency (Freq) (Supplementary Fig. S10).

Amino acid change (protdesc) was recognized as the most important feature, followed by variant allele frequency (Feq) and minor allele frequency (MAF) (Supplementary Fig. S3). The distribution of minimal depth (Supplementary Fig. S11) denotes the first time this variable (feature) is used to split the tree. The more important variables have lower min depth values. The splits that cause the larger increases in purity occur early, so the important variables are split early.

### Random forest routine implementation and impact on our workflow

**Figure 4:**
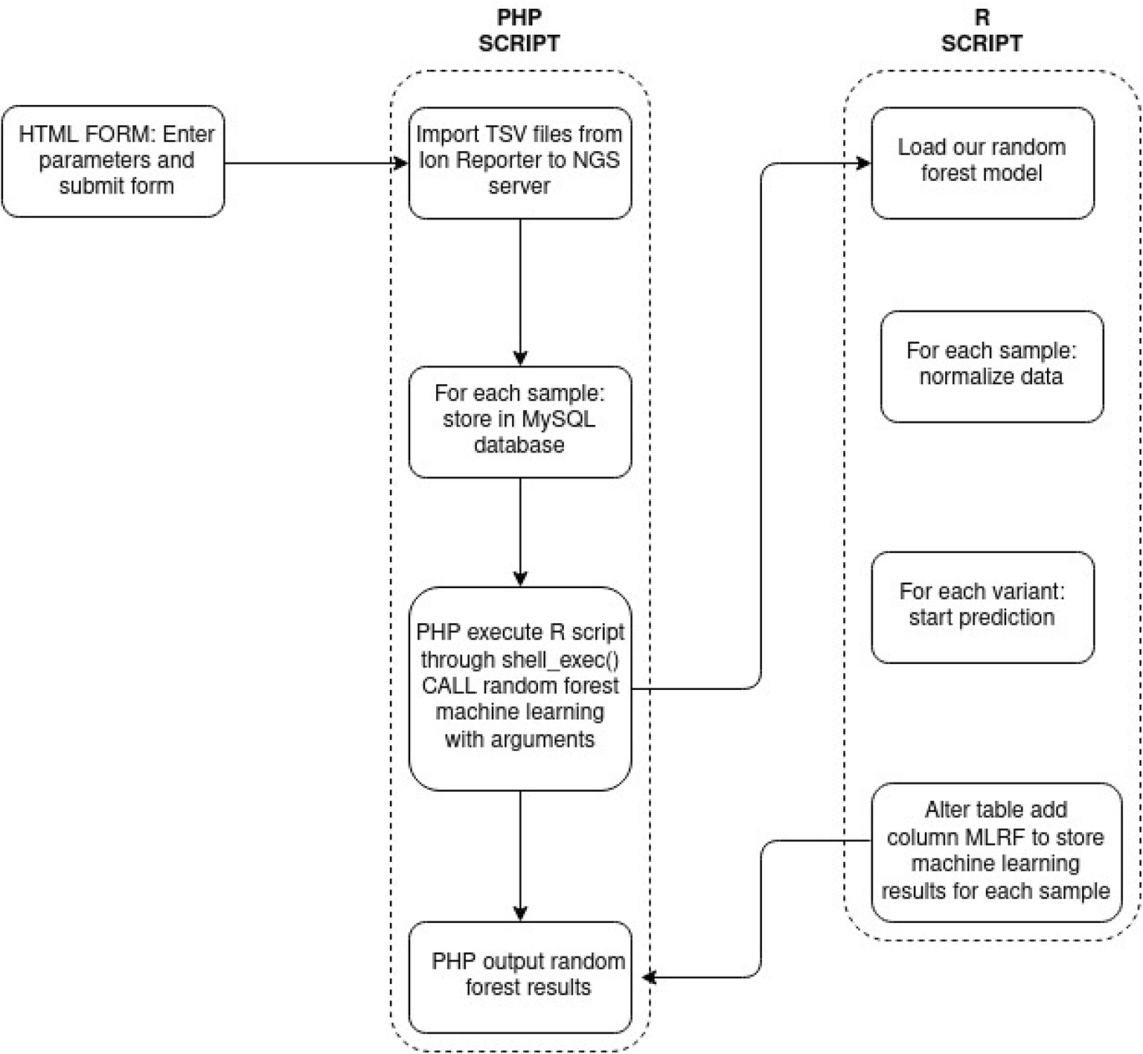
Machine learning data processing. It shows how data are imported and analyzed by random forest machine learning. This architecture was implemented in our laboratory for each NGS run.

From March 2020 until June 2021, we used RF in the NGS analysis routine at each variant imported into our MySQL database. The architecture of our information system was developed in Hypertext Preprocessor (PHP), so it used R script to call machine learning at each run (Fig. 4).

The NGS database of the first 2 months of the year represented more than 2000 variants. The RF model misclassified 20 variants (which represents an error rate of < 1%). The errors were nomenclature problems or false positives. We found 2 errors about the same MET mutation in the splice site.

### Testing neural network on our dataset

We tried several machine learning algorithms, but it may be interesting to determine whether neural networks can achieve a good recognition pattern on our dataset. We will use NeuralNet R package version 1.44.2.

Our aim is to connect the 7 input features (chr, POS, exon, Freq, MAF, coverage and protdesc) to the correct output class (YES, NO) by using a fully connected artificial neural network. We will use a 10-fold cross-validation (10-CV) method on our ANN.

Neural networks, commonly known as artificial neural networks (ANNs), are simple imitations of the neuron functions in the human brain for solving machine learning problems. As we did with machine learning, for our “system” to be able to compare these variants, we must give our neuron network a multitude of pathogenic variants and benign variants by indicating what each variant represents. In the learning phase, the ANN analyzes each of the data and draws its result. The goal, once the learning phase is finished, is that the system will be able to predict whether a variant is pathogenic with a certain probability. The neural network is fully connected, which means that all nodes (neurons) are connected, which is a set of neurons organized in layers and interconnected by “weights”. Each weight is a value that is used to spread information, and they are the base of our networks, which allows all learning processes to be performed. The first layer is called the input layer, and the last layer is called the output layer. In our study case, we have 7 inputs and 2 outputs. All layers between the input layer and the output layer are called hidden layers. The number of hidden layers and nodes is very inconstant, potentially from zero to infinity. There are only empirical rules to define how many hidden layers and nodes are needed. As every problem is different and each network approximates a system differently, there are no precise rules or even a mathematical formula that allows us to determine which numbers are appropriate for that specific problem^13^ . Despite this fact, we can use an optimum choice of the number of neurons:

- A small number of neurons will lead to high error for the system, as the predictive factors might be too complex for a small number of neurons to capture^13^
- A large number of neurons will overfit to the training data and not generalize well^13^
- The number of neurons in each hidden layer should be somewhere between the size of the input and the output layer, potentially the mean^13^
- The number of neurons in each hidden layer should not exceed twice the number of input neurons, as the system is probably grossly overfit at this point^13^

There are many rule-of-thumb methods for determining the correct number of neurons to use in the hidden layers, such as the following. The number of hidden neurons should be between the size of the input layer and the size of the output layer. The number of hidden neurons should be 2/3 the size of the input layer, plus the size of the output layer. The number of hidden neurons should be less than twice the size of the input layer^12^.

The first step is to give understandable information to our input layer: numbers. The information will propagate layer by layer, and each neuron will react with a numerical value based on its incoming connections, according to their weights. This will define their activation rates.

These neurons receive several entrances from input neurons $\left[ a_{0},a_{1},a_{2},a_{3},a_{4},a_{5},a_{6},a_{7}\right]$ and produce one output value. Each of these entrances is balanced by one weight $w_{i}$. Inside this neuron, there is an activation function $\sigma$.

It is a mathematical function that takes a number as input and outputs a result. All we need to know about the activation function is that it will help us to make our input values fit in a small interval. Most of the time between 0 and 1 or between − 1 and 1.

The most frequently used functions are the hyperbolic tangent (tanh), sigmoid function, linear adjustment (ReLu), and softmax. For our ANN, we will use a sigmoid function, which is defined as:

$$\begin{aligned} \varPsi (x)=\frac{1}{1+e^{-x}} \end{aligned}$$

Please see the Supplementary Data section—Working principle of ANN for further information.

As input and output are already defined, the question is how many hidden layers and nodes do we need?

**Figure 5:**
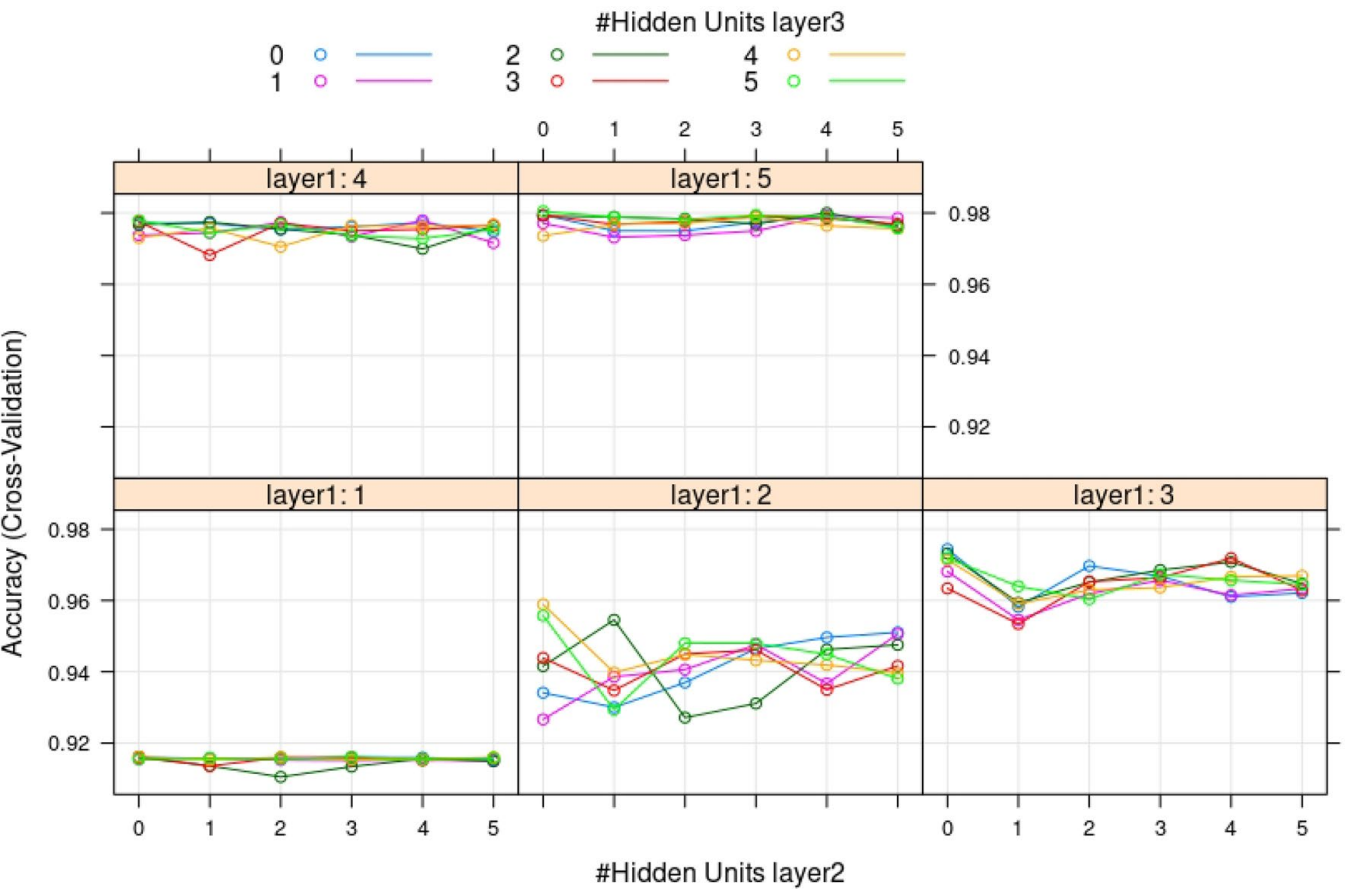
Results of accuracy (cross-validation) as a function of hidden unit layer parameters, size and number of nodes. Tune grid search with a combination of layer 1 (L1) in the range of [1,5] nodes, layer 2 (L2) in the range of [0,5] nodes, and layer 3 (L3) in the range of [0,5] nodes. The accuracy reached the highest value of 97.97% when L1 = 5, L2 = 0 and L3 = 5.

To answer this question, we will use the tuned grid method with 10 cross-validations. We will define 3 hidden layers $L_{i}=\left( L_{1},L_{2},L_{3}\right)$ and each layer contains a grid of node values: $L_{1}=[1{-}5]$, $L_{2}=[0{-}5]$, $L_{3}=[0{-}5]$. It took 22 h to perform the tune grid search against 30 min with random forest to obtain a result. After performing the 10 cross-validation tuning grid layer optimizations, the system found that the best accuracy was obtained by $L_{1}=5$,$L_{2}=0$ and $L_{3}=5$, with an accuracy of 97.94% ($\simeq$98%) and a kappa value of 0.9588 ($\simeq$0.96) (Fig. 5). sensitivity: 0.9843 and specificity: 0.9745.

Confusion matrix on the test set (Supplementary Fig. S4). Looking at the validation set results, the ANN performed well on unseen data with an accuracy of 97.91% and a kappa of 96% (Supplementary Fig. S5). sensitivity: 0.9819, specificity: 0.9763.

The AUC for the YES class label is 0.994532 and for the NO label is 0.9945321. The higher the AUC, the better the performance of the model at distinguishing between the positive and negative classes. As the AUC is close to 1, the classifier can perfectly distinguish between all the positive and the negative class points correctly. The ROC curve showed a good classifier (Supplementary Fig. S6).

**Figure 6:**
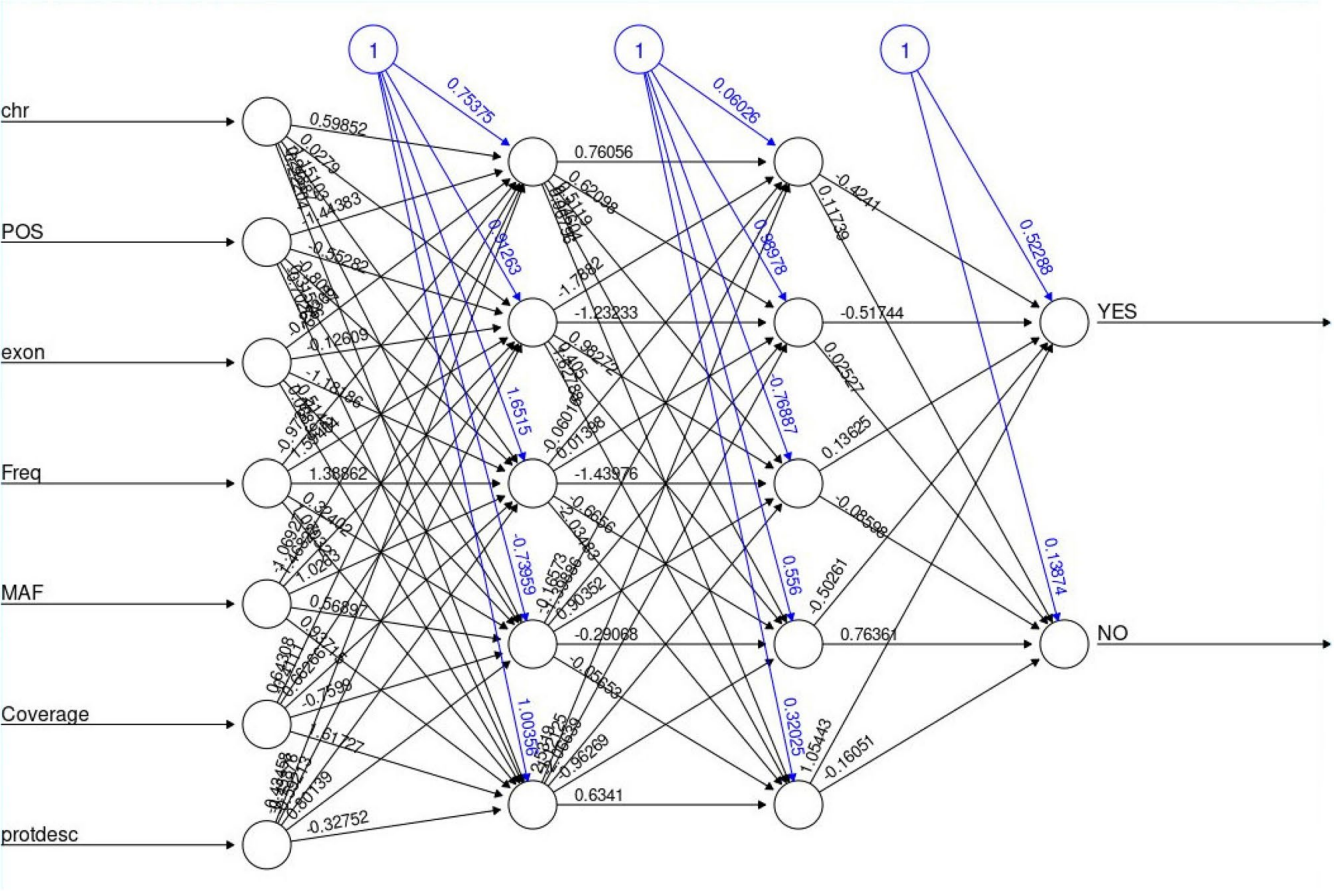
Fully connected artificial neural network (ANN) for oncosomatic variant. It is composed of 7 input features: chromosome number (chr), coordinate position of the chromosome (Pos), exon number, variant allele frequency (Freq), minor allele frequency (MAF), coverage, acid amino change (protdesc), with 2 hidden layers of 5 hidden nodes each. The output is the answer of the ANN, and the output value is YES for pathogenic and NO for benign. In binary representation, YES is coded as 1 for pathogenic and NO coded as 0 for benign.

Our neural network variant classifier has the following architecture (Fig. 6): 7 inputs + 2 hidden layers of 5 nodes each and two outputs.

## Discussion

Our aim was to use AI to interpret NGS oncosomatic analysis relying on 7 parameters: chromosome, position, exon, variant allele frequency, minor allele frequency, coverage, and protein description. We developed an accurate and efficient method based on random forest machine learning that classifies a set of variants with high precision from NGS data. Our implemented model obtains decisions and probability for each variant call and allows a biologist to accept or reject variants (Supplementary Table S1). The model was developed in the R language (R version 3.6.0, RandomForest version 4.6-14, NeuralNet version 1.44.2), which is machine learning open source modules; hence, more users can tune this model on their data by using our training database.

ML and, more particularly, the RF model has already been used in several publications as a tool for risk evaluation, quality control or predictions based on sequencing data^2,4,9^. Furthermore, the applications we found using RF rely on the analysis of VCF (variant caller) files such as Smurf, which predicts a consensus set of somatic mutation calls, or Octopus, which uses BAM files as a training dataset to classify variant calls^9,10^.

Our tool classifies oncosomatic variants when already annotated (see Supplementary Data Fig. S7). It is effortless to implement in an information system and user-friendly for “noncomputer” science persons. Moreover, our tool is not intended to replace biologists but is rather an aid for decisions facing the multitude of somatic variants present at each NGS run analysis. To our knowledge, it has never been published for routinely interpreting NGS analysis. Our RF model shows excellent results with an error rate of < 0.5% since we routinely use it. Errors are nomenclature errors and false positives absent from our training database. Nomenclature errors are difficult to correct because the ion reporter annotates variants automatically, so our error rate can never be zero, and we have to stay vigilant to spot them. We can manually change them if there is a nucleotide error, for example, after our RF model is correct. Nevertheless, we cannot eliminate read errors from sequencing, and we easily improved false positive errors. At every new false positive mutation, we increment our training database, so that our RF model can be improved at each NGS run. ML is the oldest and simplest technology. It is based on an algorithm that adapts the system, based on feedback from humans. Implementing this technology implies the existence of organized data. The system is then fed with structured and categorized data, allowing it to understand how to classify new similar data. Based on this classification, the system then performs programmed actions. After the first phase of use, the algorithm is optimized based on a tuned grid search of two parameters (number of trees: ntree and number of variables randomly sampled at each split: mtry), which informs the system of incorrect classifications and indicates the correct categories. ML works from a controllable database, which means that at each moment, we can modify the training database to correct prediction errors. The system must have many more entries to achieve reliable results. We also have an error rate of < 0.5% for our glioma panel (AmpliSeq glioma custom panel: AKT1, ATRX, BRAF, CDKN2A, CIC, EGFR, FGFR1, H3F3A, HIST3B, IDH1, IDH2, NOTCH1, PIK3CA, PIK3R1, PTEN, PTPN11, TERT, TP53) NGS analysis. We chose RF as the first model for several reasons. It is easier to set up, since it combines regression and classification, which saves time on data preparation. Additionally, there is a lower chance of “overfitting” (cases where the model is perfectly adapted to data, but does not generalize very well). RF achieves more precise predictions but does not need as much pruning. Indeed, we have a multitude of random trees using random features, and thus, the individual trees are strong but not so correlated with each other. Pruning is a method used to prevent overfitting decision trees by trimming certain levels. Routinely implementing RF is very easy and fast. The processing time for one chip of 32 samples takes less than 1 min to predict each variant. RF has already been tested and used by other authors for NGS analysis. It seems to be a good reproducible model for this type of interpretation^2,5,6,11^. Moreover, the random forest algorithm is a machine learning algorithm that runs efficiently on large datasets with high accuracy^15^. Random forest was successfully applied in other laboratories to predict and classify genomic data analysis, and it is an appropriate tool for predicting clinical outcomes under high-throughput genomic platforms^23^. Random forest in comparison with k-nearest neighbors (KNN), linear discriminant analysis (LDA), classification and regression trees (CART), support vector machine (SVM) performed better than the others algorithms in terms of the error in prediction. Lee et al.^24^ showed that RF has the best performance compared to all tree-based methods. A machine learning method based on the random forest for predicting the classification of variants was also successfully applied in medical case diagnosis of breast cancer^25^. They built a computer “help” diagnostic system to determine if a breast tumor is benign from malignant tumors. They obtained 99.82% classification accuracy in the test phase, and their results can be applied to other breast cancer problems. Their proposed method was based on N-fold cross-validation with a phase one where RF was trained and tested on the training set and validation set from the University of California, Irvine Breast Cancer Wisconsin (Diagnostic) dataset. Lai et al.^26^ developed an interesting machine learning method based on the random forest to predict the classification of variants in genes associated with high risk for hereditary cancer. A training base of 14,226 missense variants in genes associated with high risk for hereditary cancer and 5398 missense variants with elevated risk for cardiovascular disorders were used to train the model. They managed to classify missense variants with a high precision of 98.3% AUROC by using 10-fold cross-validation. In our study, we also attempted to determine whether any neural network can achieve a good somatic classifier, and it showed the ability to classify mutation variants, but tuning a neural network takes time when another approach might be better and save considerable time. Considering using a different ML algorithm to ANNs—decision trees based on approaches such as random forest can be faster and more effective than deep learning on many problems. However, they also have the capacity to learn subtle differences from large quantities of data where simpler algorithms may reach a limit. Neural networks have provided good accuracy in the prediction of variant mutations. We compared the performances of the neural network and random forest on unseen data, and RF was slightly over in accuracy most of the time in comparison with the neural network. However, ANN shows the ability to adapt to new data where RF failed. For example, the two MET mutations in splice sites missed by RF were well predicted by the neural networks as pathogenic (Supplementary Table S1). After modifying our database, our RF model achieves a correct classification. It is always a good idea to run ANN in parallel and compare results with RF. A study in the optimization of the number of hidden layers and neurons can be a good approach for a future study, particularly for the predictions of the pathogenicity of the BRCA1/BRCA2 genes. They present more parameters in the interpretations of pathogenicity. Despite ANN’s good predictions, we decided to routinely implement the RF because the classifier showed excellent prediction values. The final trained model achieved an accuracy of 99.77% and an ROC-AUC of 0.99, and we were close to a perfect classifier. Our random forest and artificial neural network models can be implemented to other laboratories if they use Ion Reporter to annotate and extract variants in tabulated separated values (.tsv) file format. The file exported from Ion Reported must have the same 7 input features (chromosome, position, exon, variant allele frequency, minor allele frequency, coverage and amino acid change) and be imported through the application to start predictions on the variants. It should be clear that after implementation in another laboratories/city/country, the performance would still need to be evaluated to see if the model generalizes to the new conditions which may involve for example different protocols and samples of different characteristics.

## Conclusion

We presented a machine learning method to classify oncosomatic variants detected by NGS on lung, colorectal, melanoma and glioma tumors. RF is a great AI algorithm to produce a predictive model for classification problems. It is easy and fast to implement in an information system, and it does not need any powerful machine to perform. Our model was optimized to use the lowest amount of computer resources, making it faster even on average computers. The only disadvantage is the high number of trees that can make the computational process much slower. Since we used it routinely, our RF model shows good results with the ANN in parallel. It can easily be implemented in other molecular biology laboratories and become a true aid in analysis in routine NGS oncosomatic interpretation. AI is taking an increasingly important place in molecular biomedical analysis and can be beneficial in processing large quantities of medical data.

## Supplementary Information

Supplementary Information.
