# Supplementary material for: Machine learning random forest for predicting oncosomatic variant NGS analysis
Source: Sci Rep. 2021 Nov 8;11:21820. doi: 10.1038/s41598-021-01253-y (PMC8575902; doi:10.1038/s41598-021-01253-y)
Supplement: Supplementary file 1 — Supplementary Information. [file 41598_2021_1253_MOESM1_ESM.pdf]

Supplementary data

Working principle of artificial neural network (ANN)

On artificial neural network, each neuron works in three steps:

Initially, we will start by generating a weighted average of the inputs, then we multiply by their corresponding weights.

$$\sum (a_i \times \omega_i)$$

At that point, we will include what we call a bias b. It is a specific value for each neuron.

$$\sum (a_i \times \omega_i) + b$$

Finally, we have a result that we can send to our activation function σ

$$\sigma \left(\sum (a_i \times \omega_i) + b \right)$$

In matrix notation:

$$a^{(1)} = \sigma \left(W a^{(0)} + B \right)$$

$a^{(0)}$ represents the first layer, W the weights relating the first layer to the first neurons of the second layer. $a^{(1)}$ the vector result of second neural layer. B is the vector which contains the bias' value for every neuron. We apply this formula to all others layers (Figure S11).

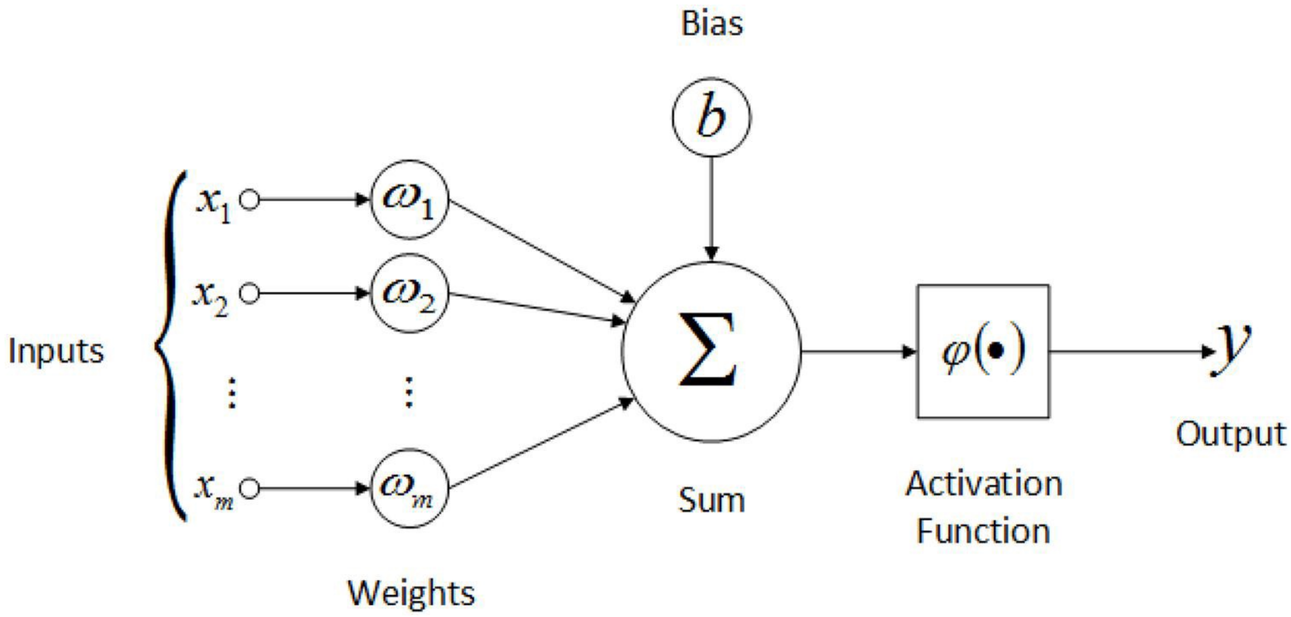

Figure S11. Artificial Neural Network (ANN)

The bigger the given value in the input, the more the output value will be close to 1. On the other hand, the more the input value is negative, the more the exit value will approach zero. It is this result which informs us how the neural network is activated. At 0, we will say the neurons are inactive, at one it will be fully activated and can have all the intermediate states. To increase control on this exit value we bring the bias b. Bias is the threshold where we will consider the neuron activated. For example, if our sigmoid function returns a 50% activated neuron when it receives 0 input, if we add a bias to our equation, the neuron will be activated more easily. This value allows us to shift the activation function. We will include the bias b to the input value of our function. This value will be adjusted at the same time as all of our weights during the network training phase.

After this calculation, we end up with an exit value. This process, where our value is propagated until delivering a result, is called feedforward. Information about the initial variant will propagate through the hidden layers based on weight values.

We ultimately obtain a result in the output layer. According to our model, we have 2 output neurons. Each of them is the representations of a number between zero and one (NO or YES).

The degree of activation of each of these neurons represents the percentage chance that the variant passed as input is pathogenic -> 1 (YES) or 0 (NO) (according to our training dataset).

For example, variant chr12:25398280/ KRAS / 24.41% / 1962 / c.35G>T / p.Gly12Val, the best possible answer would be to obtain all of the neurons = NO (=0) shut off except the one representing the YES (=1).

At the beginning, our network is trying to obtain the most effective solution for the first time (First epoch:is when an entire dataset is passed forward and backward through the neural network only once) all of its output neurons will be more or less activated, it predicted randomly because he had no information about the result we were expected. To improve the result, we have to correct the parameters of our neural network. The sole way to alter the behavior of our network is to vary its weights. The learning process consists in achieving the most exact possible weights to provide an accurate answer. For this, we compare the given result and the predicted result.We will end up with a value called loss. The higher the loss, the farthest, the network is from the precise answer. With the loss, we are capable to deduct which weights have participated the most on our incorrect answer.

At that point we have to adjust the weights to minimize the loss and get closer to the precise answer. On the next epoch, we try with another variant. The network will be wrong again, but it is closer to the expected answer. Loss value is a representation of our error rate on this network for each of its output neurons. Then amongst our parameters, we will have to identify which combination of each of these weights will allow us to obtain the most insignificant possible loss. To evaluate all these values, we will

implement the algorithm of gradient descent (optimizer). Gradient descent uses derivatives of a function. As we know, the derivative function allows us to know the tangent's gradient of the curve for a given point.

According to that tangent, we will be capable to determine how to change the entry weight to get closer to the result that we are looking for. If the derivative is positive, we will have to lower down our weight and vice versa. We can also deduct that the more important our derivative is, further we would be from the accurate result. We will have to increase our input, recalculate the derivative and restart those steps again until we reach a nil derivative. Which means we reached the minimum loss.

Gradient descent contains a parameter named learning rate. Initially, the steps are more substantial. That means the learning rate is higher and as the point goes down the learning rate becomes smaller because the steps get shorter. The backpropagation system corresponds to the action of correcting the weights that led to the error, doing it layer by layer implementing the gradient descent.

We merely have to duplicate these fitforward and back propagation steps again and again until we obtain the optimal result. We repeat these steps thousands of times with many somatic variants and adjust the weights each time. After many epochs, the network is capable to recognize oncosomatic variants. Progressively, the network will be effective to have its own representation of each variant introduced into the inputs. After the training process, we can test the network on mutation variants that we have never seen until now. If the learning is done correctly, the network delivers us our expected answer. All these calculation processes are automated thanks to NeuralNet package.

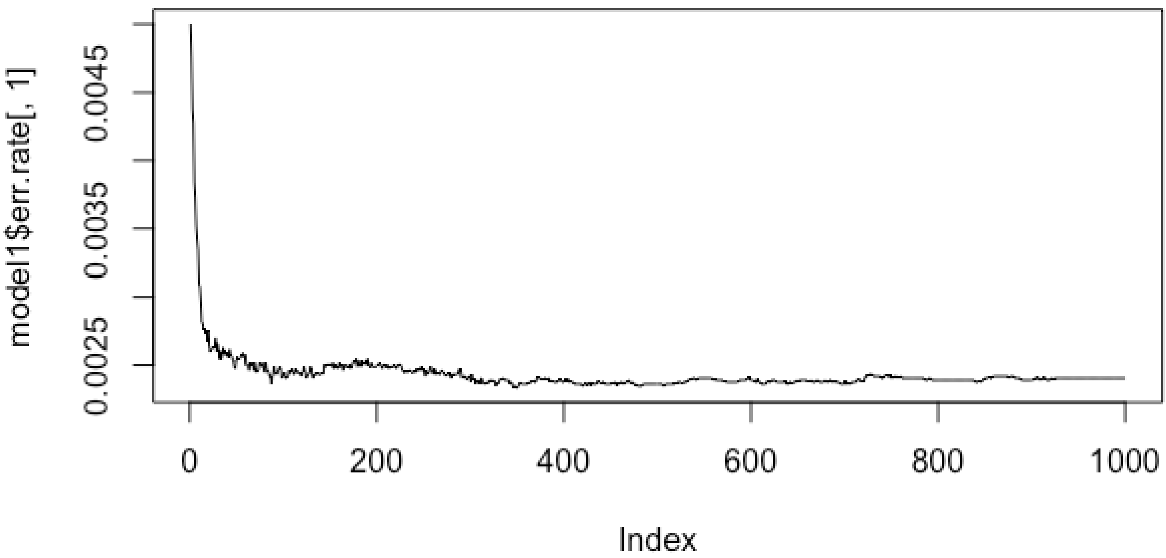

Figure S1 : Error rate output (out of bag) of random forest (model1$err.rate[,1]) in function of number of trees (Index). The plot shows that the best error rate was stabilized for ntree = 500. All values before 500 oscillate excessively and after it's more machine time consuming. The number of trees in random forest to be used for feature selection is defined by the lowest error rate in the initial random forest of 500 trees with 7 variables (chromosome, position, exon, variant allele frequency, minor allele frequency, coverage, amino acid change) randomly sampled as candidates at each split. Out of bag (OOB) error rate to assess the quality of random forest prediction of oncosomatic variants shown as a function of the number of decision trees generated during the machine learning with mtry=4.

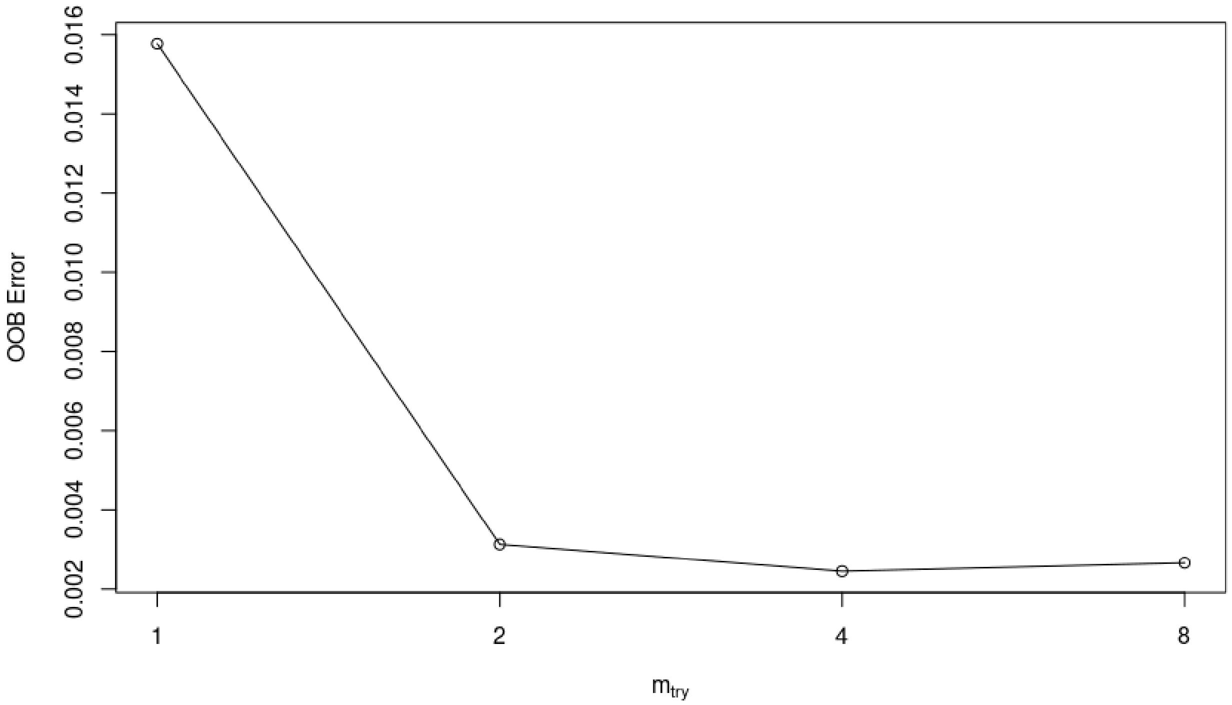

Figure S2. Optimal number of variables for each split (mtry) is reached for mtry=4, where the model as the lowest out of bag (OOB) error. TuneRF R function was used to search the best optimal value of mtry. Parameters of TuneRF: ntree: number of trees to be grown for tuning = 500, stepfactor: this is the value by which the chooses of mtry gets inflated or deflated = 0.5, improve argument specifies the improvement in OOB error value for the search to continue = 0.05

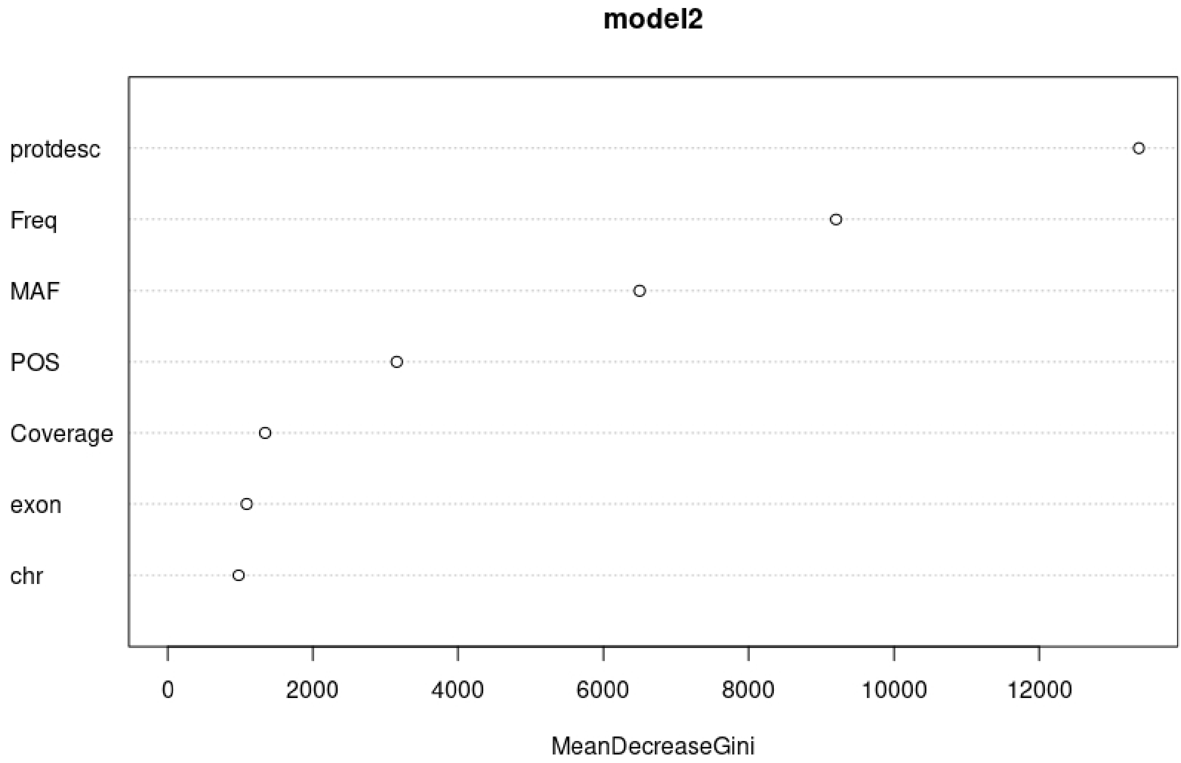

Figure S3. Features importance shows the importance of each variable chromosome (chr), exon, coverage, position (POS) minor allele frequency (MAF), variant allele frequency (Freq) and amino acid change (protdesc) in the model built. Higher MeanDecreaseGini higher the importance the variable has in the model based on Gini index. It represents how much removing each variable reduces the accuracy of the model.

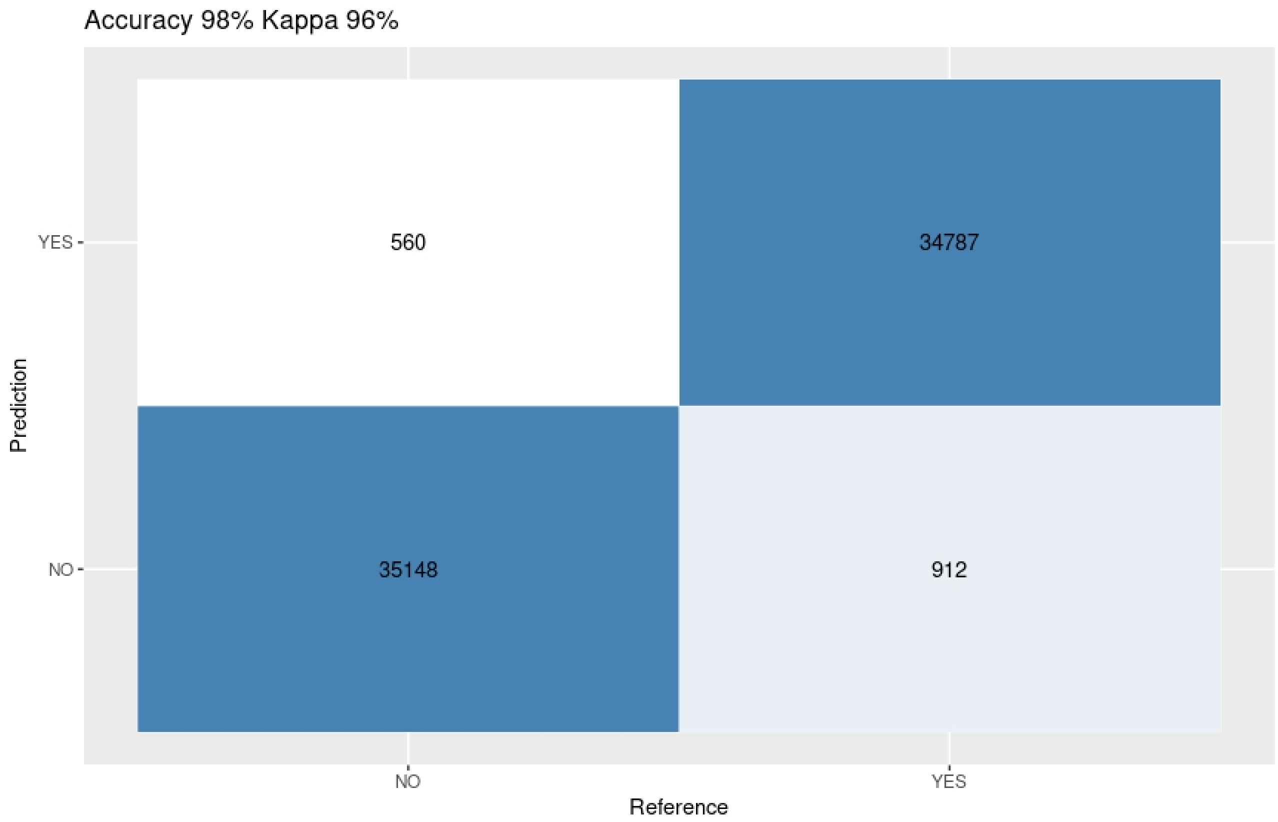

Figure S4. Confusion matrix on Test Set obtained with the artificial neural network (ANN) with the architecture of 2 hidden layers of 5 nodes each.

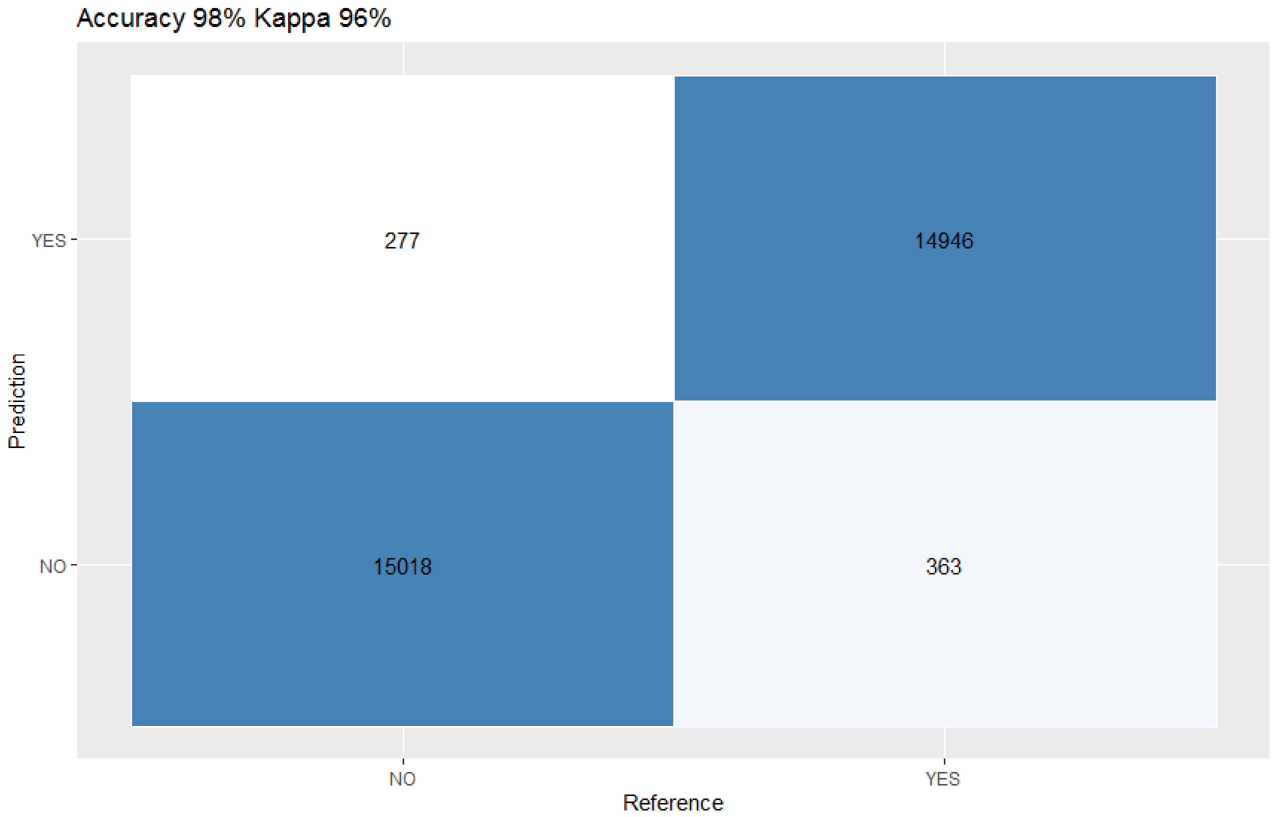

Figure S5. Confusion matrix on validation set (unseen data) obtained with the artificial neural network (ANN) with the architecture of 2 hidden layers of 5 nodes each.

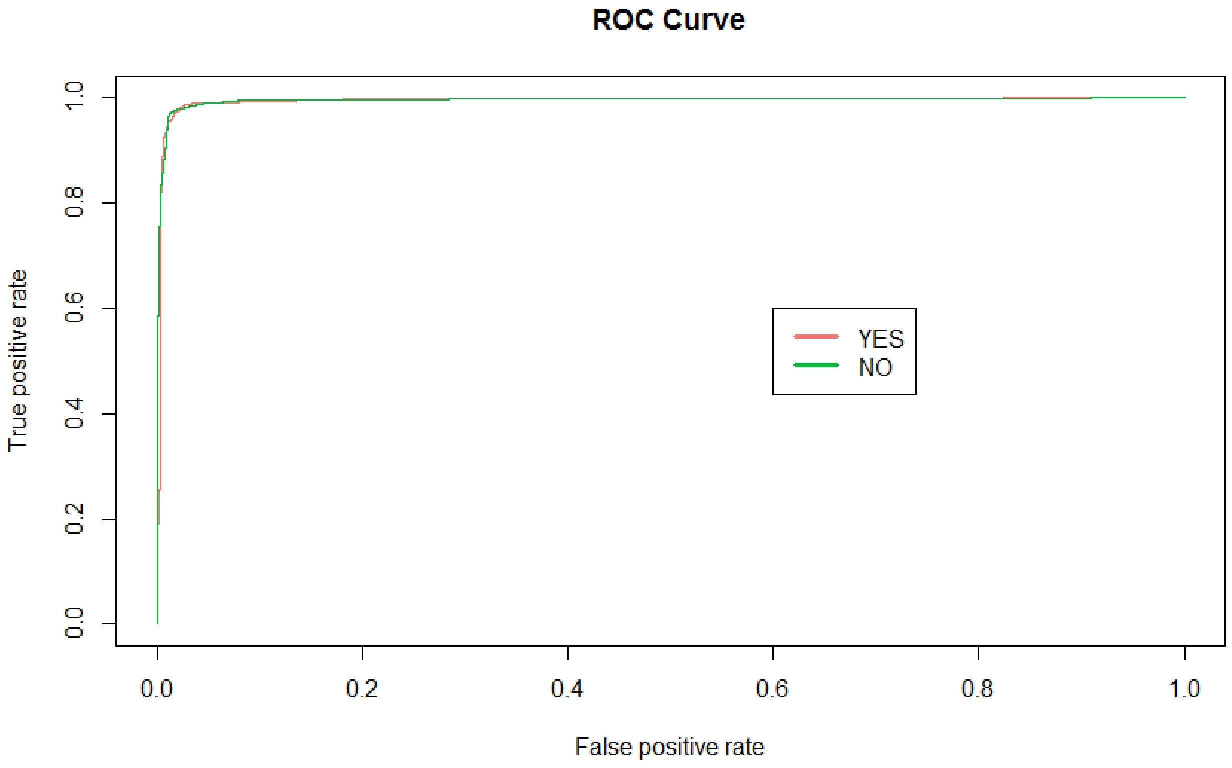

Figure S6. ROC curve for validation set obtained with the artificial neural network, composed with 2 hidden layers of 5 nodes each. 10-cross validations were assessed using with 7 functional features: chromosome (Chr), position (POS), exon, variant allele frequency (Freq), minor allele frequency (MAF), coverage and amino acid change (protdesc) for the model artificial neural network for oncosomatic variants. Model performance was evaluated using receiver operating curve (ROC). The area under the curve is denoted AUC. AUC obtained was 0.99. A large area under the curve was observed. The model could then determine whether a variant was benign or pathogenic with a minimal error rate

id	isMut	VarClass	MLRF	Location	Exon	Coding	ProtDesc	MAF	Coverage	%Frequency	TP53-IARC	ClinVar	Locus	PHRED_Q
1			NO	DDR2:exonic:NM_006182.2	13	c.1509C>T	p.(=)	0.0	2000	51.30			chr1:162741818	41
2			YES	DDR2:exonic:NM_006182.2	14	c.1750G>A	p.Gly584Arg		2000	24.30			chr1:162743280	36
4			NO	ERBB4:intronic:NM_005235.2	0	c.421+58A>G	p.?	0.355	2000	58.15			chr2:212812097	41
5			YES	PIK3CA:exonic:NM_006218.3	10	c.1636C>A	p.Gln546Lys		1979	27.39		Likely pathogenic,Pathogenic	chr3:178936094	38
6			NO	FGFR3:exonic:NM_000142.4	14	c.1953G>A	p.(=)	0.044 (ref)	879	99.66			chr4:1807894	42
7			NO	PDGFRA:exonic:NM_006206.4	12	c.1701A>G	p.(=)	0.042 (ref)	2000	99.60		Benign	chr4:55141055	45
8			NO	EGFR:intronic:NM_005228.3	0	c.1498+22A>T	p.?	0.227 (ref)	1999	10.91			chr7:55228053	29
9			NO	EGFR:intronic:NM_005228.3	0	c.1498+29G>A	p.?		2000	13.35			chr7:55228060	31
10			NO	EGFR-AS1:exonic_nc:NR_047551.	20	c.2361G>A	p.(=)	0.433	2000	2.20		Benign,Likely benign	chr7:55249063	8
11			NO	MET:exonic:NM_001127500.2	2	c.534C>T	p.(=)	0.088	1987	38.10		Benign	chr7:116339672	39
12			NO	MET:exonic:NM_001127500.2	20	c.3912C>T	p.(=)	0.352	1989	62.95		Benign	chr7:116435768	42
13			NO	RET:exonic:NM_020975.4	11	c.2071G>A	p.Gly691Ser	0.169	872	46.56		Benign,Likely benign	chr10:43610119	36
14			NO	RET:intronic:NM_020975.4	0	c.2136+49C>T	p.?		843	26.33			chr10:43610233	32
15			NO	RET:exonic:NM_020975.4	13	c.2307G>T	p.(=)	0.287 (ref)	1998	48.30		Benign	chr10:43613843	40
16			NO	RET:intronic:NM_020975.4	0	c.2608-24G>A	p.?	0.173	1167	4.11		Benign	chr10:43615505	18
17			NO	RET:exonic:NM_020975.4	15	c.2712C>G	p.(=)	0.172	1166	42.88		Benign,Likely benign	chr10:43615633	37
18			YES	RET:exonic:NM_020975.4	16	c.2767C>T	p.Leu923Phe		1959	24.60			chr10:43617430	36
20			YES	TP53:exonic:NM_000546.5	8	c.848G>C	p.Arg283Pro		1918	27.22	deleterious		chr17:7577070	38
21			YES	TP53:exonic:NM_000546.5	7	c.741_742delCCinsTT	p.Arg248Trp		1942	31.67			chr17:7577528	38
22			NO	TP53:exonic:NM_000546.5	7	c.741C>T	p.(=)		2000	31.10	NA	Likely benign	chr17:7577540	39

Figure S7. Example of NGS analysis result with Ion interface and the MLRF (machine learning random forest) prediction result on variant.

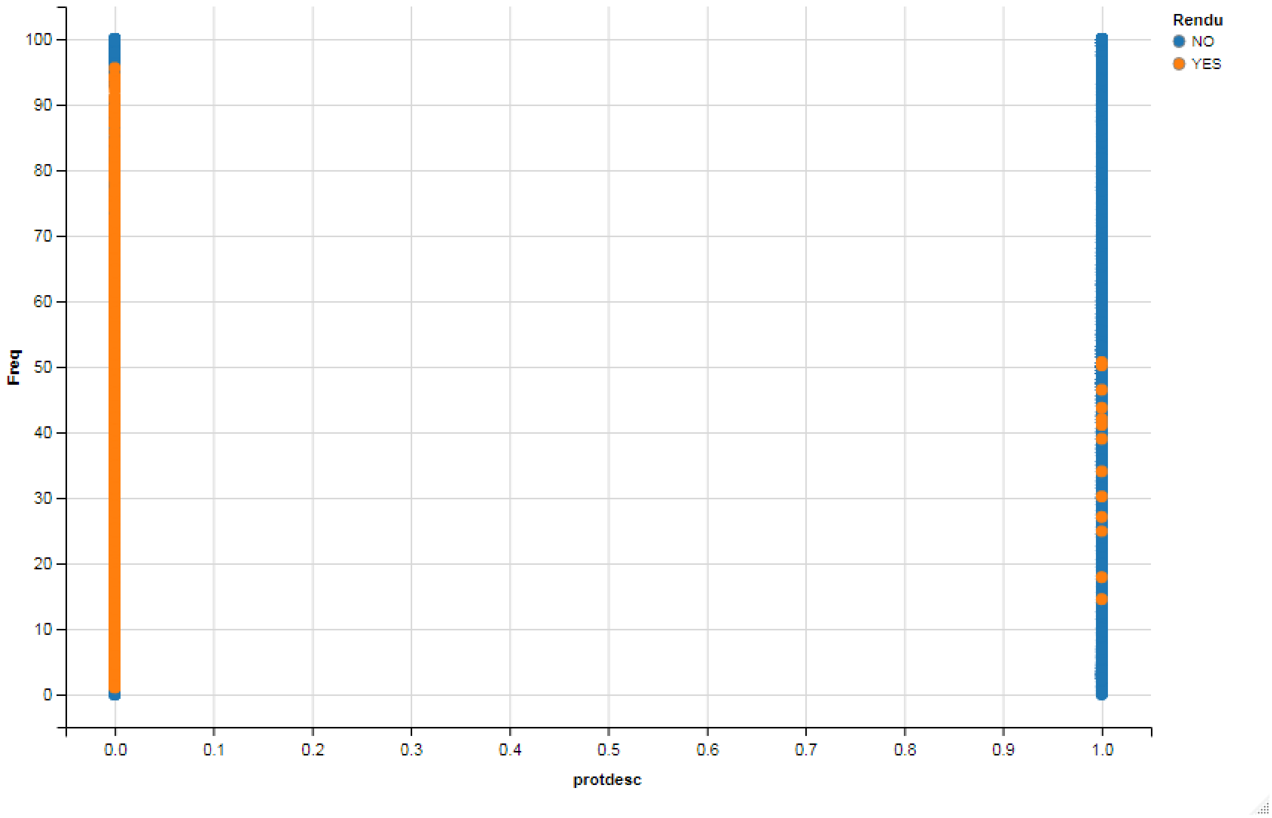

Figure S8. Variant allele frequency (Freq) in function of the amino acid change (protdesc). A strong proportion of variants were flagged as pathogenic (labelled as RENDU = YES = 1) by the biologist when the amino acid change is different from silent p.(=) (protdesc $\neq 1$).

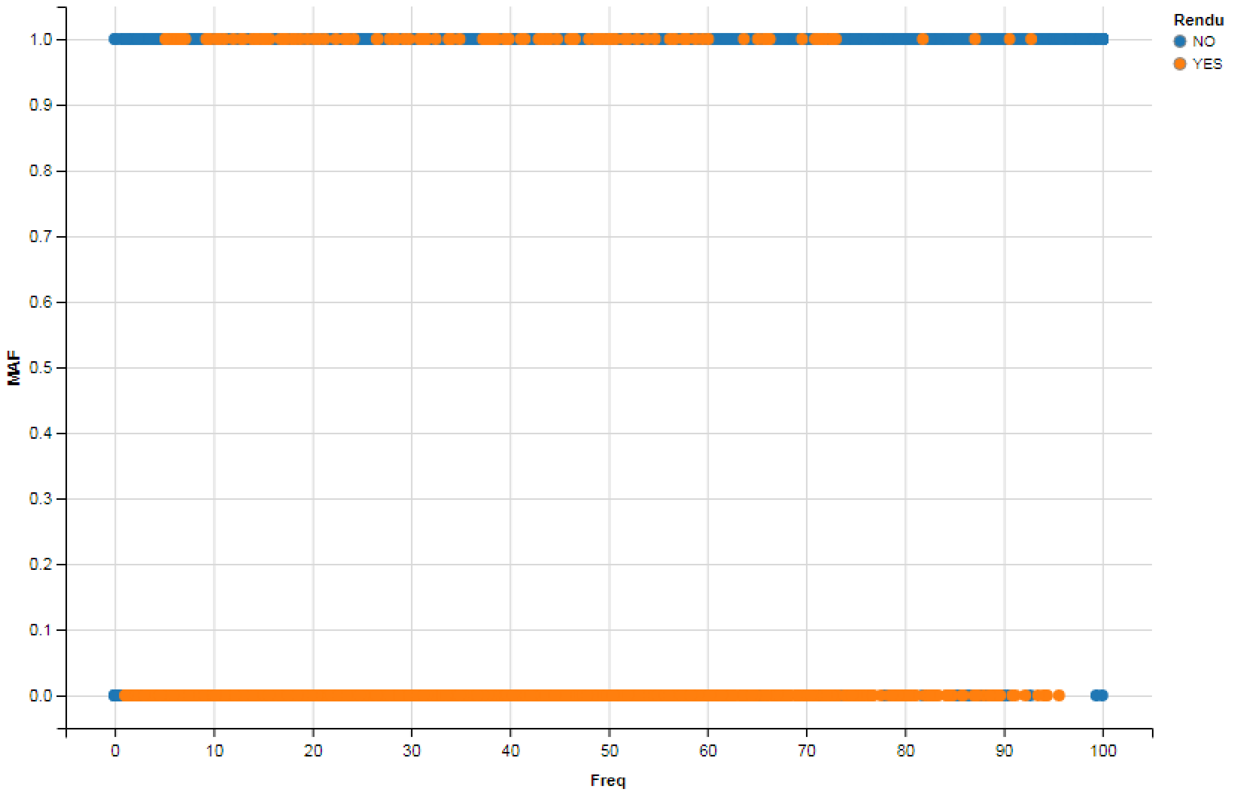

Figure S9. Minor allele frequency (MAF) in function of variant allele frequency (Freq), we can observe more variants were flagged as benign (RENDU = NO) when a MAF was present on a range of Frequency [90;100%].

Table S1. Random forest (MLRF) decision with probability versus artificial neural network (ANN) decision on NGS variants. Green colour means in accordance with biologist decision and in red not in accordance.

Genes	Coding	Locus	Type	Exon	VAF	MAF	Coverage	Protdesc	Biologist Decision	MLRF	MLRF proba	ANN	ANN Prob
KRAS	c.34_35delGGinsTT	chr12:25398280	MNV	2	43.19	0	3941	0	YES	YES	1.000	YES	0.998280525
KRAS	c.34G>T	chr12:25398280	SNV	2	25.47	0	3957	0	YES	YES	1.000	YES	0.998280525
ERBB4	c.955G>T	chr2:212578302	SNV	8	12.98	0	4000	0	YES	YES	1.000	YES	0.996720195
TP53	c.592G>T	chr17:7578256	SNV	6	50.43	0	3881	0	YES	YES	1.000	YES	0.998146176
KRAS	c.35G>C	chr12:25398280	SNV	2	10.19	0	3965	0	YES	YES	1.000	YES	0.998280466
EGFR	c.2240_2254delTAAGAGAAGCAACAT	chr7:55242467	INDEL	19	7.58	0	3877	0	YES	YES	1.000	YES	0.964137554
TP53	c.701A>G	chr17:7577578	SNV	7	56.12	0	3981	0	YES	YES	1.000	YES	0.997943997
SMAD4	c.1587dup	chr18:48604764	INDEL	12	30.09	0	2888	0	YES	YES	1.000	YES	0.984070003
NRAS	c.182A>G	chr1:115256528	SNV	3	48.26	0	3997	0	YES	YES	1.000	YES	0.996720195
POLE	c.1348G>A	chr12:133250172	SNV	13	53.33	0	1999	0	YES	YES	1.000	YES	0.994729102
EGFR	c.2308_2309insCCAGCGTGG	chr7:55248998	INDEL	20	64.28	0	1982	0	YES	YES	1.000	NO	0.815051854
TP53	c.749C>T	chr17:7577528	SNV	7	49.13	0	1964	0	YES	YES	1.000	YES	0.983990073
MET	c.2942-19TCTTTCTCTCTGTTTTAAGA>T	chr7:116411884	INDEL	14	6.14	0	1943	1	YES	NO	0.557	YES	0.995143354
TERT	c.1-124C>T	chr5:1295228	SNV	0	49.90	0	1950	1	YES	YES	1.000	YES	0.998228788
FBXW7	c.1513C>T	chr4:153247289	SNV	10	17.08	0	1997	0	YES	YES	0.998	YES	0.996720195
KRAS	c.436G>A	chr12:25378562	SNV	4	27.70	0	2000	0	YES	YES	1.000	YES	0.998292804
NRAS	c.182A>T	chr1:115256528	SNV	3	69.93	0	1992	0	YES	YES	1.000	YES	0.996720195
ERBB4	c.421+58A>G	chr2:212812097	SNV	0	40.56	1	3994	1	NO	NO	1.000	NO	0.575408518
FGFR3	c.1953G>A	chr4:1807894	SNV	14	99.51	1	2060	1	NO	NO	1.000	NO	0.817090154
MET	c.1124A>G	chr7:116340262	SNV	2	38.65	1	3997	0	NO	NO	1.000	NO	0.575388730
HRAS	c.81T>C	chr11:534242	SNV	2	39.30	1	257	1	NO	NO	1.000	NO	0.996798575
EGFR	c.1498+22A>T	chr7:55228053	SNV	0	99.95	1	3989	1	NO	NO	0.982	NO	0.993650138
TP53	c.215C>G	chr17:7579472	SNV	4	49.46	1	3999	0	NO	NO	1.000	YES	0.983942688
MET	c.3313G>T	chr7:116415165	SNV	15	48.68	0	3991	0	YES	YES	1.000	YES	0.996720195

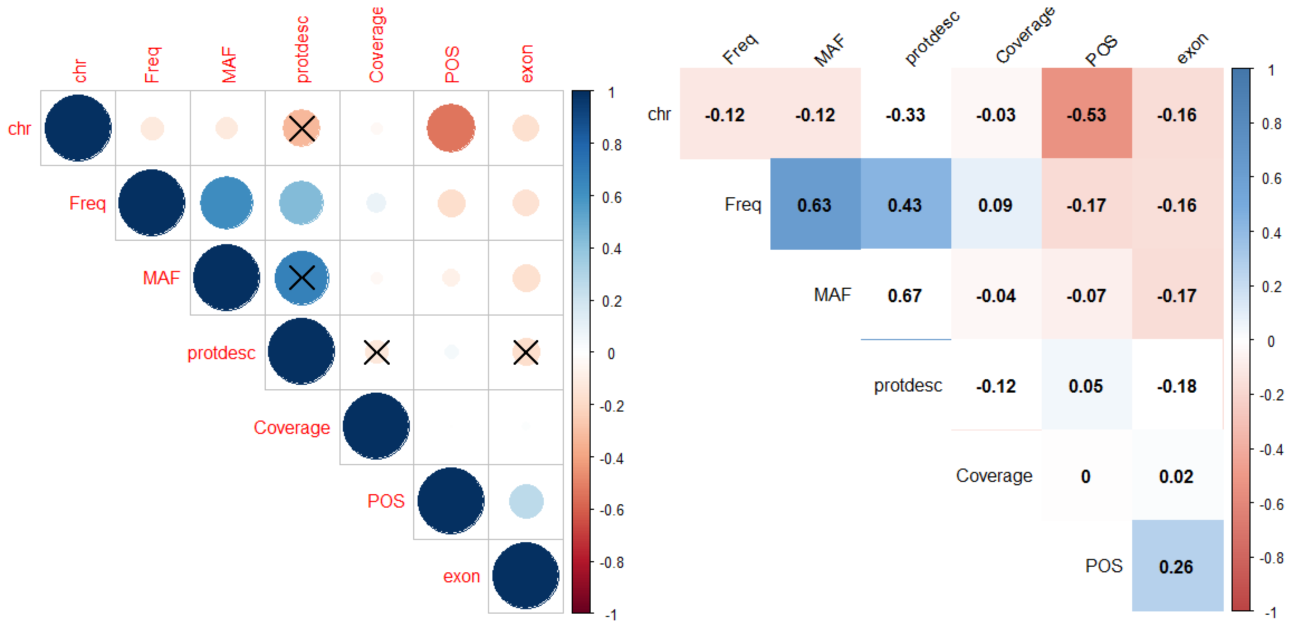

Figure S10. Correlation features between the 7 input features: chromosome (chr), variant allele frequency (Freq), minor allele frequency (MAF), amino acid change (protdesc), coverage, position (POS), exon. **Correlations** with **p-value** > 0.01 are considered as insignificant. In this case the **correlation coefficient values** are left blank or crosses are added.

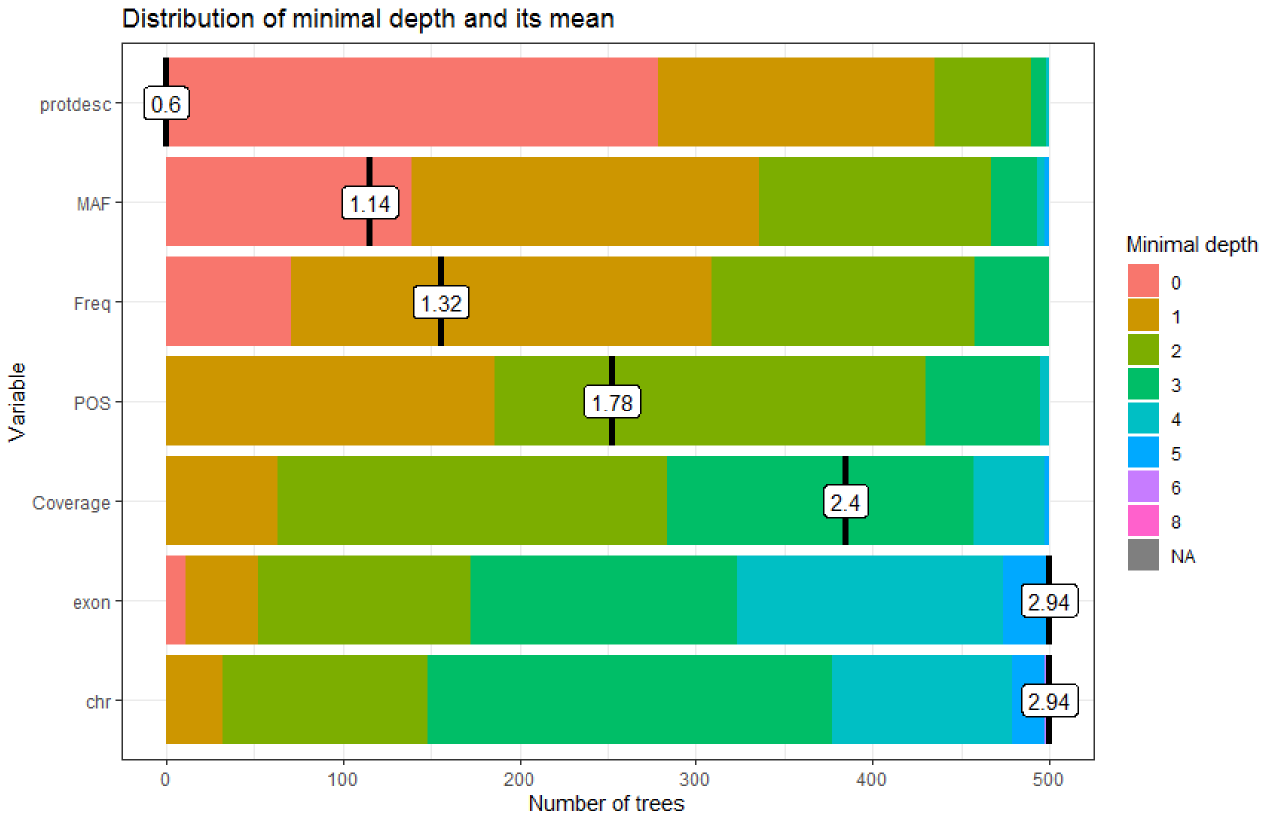

Figure S11. The smaller the mean minimal depth, the more important the variable is and the higher up the y-axis the variable will be. The rainbow gradient reveals the min and max minimal depth for each variable. The bigger the proportion of minimal depth zero (red blocks), the more frequent the variable is used for splitting trees. The range of the x-axis is from zero to the maximum number of trees for the feature. chr: Chromosome, MAF: minor allele frequency, POS: position, coverage, exon, protdesc: amino acid change.

Pipeline 2 parameters :

Alignment is done on Fastq files with bwa mem :
bwa mem genome-hg19.fa reads.fastq | samtools sort -o output.bam
samtools mpileup -A -s -O -B -f genome-hg19.fa output.bam > output.pileup

Variant Calling with Varscan2 :
java -jar Varscan.v2.4.3.jar mpileup2cns output.pileup –variants –min-var-freq 0.01 –min-avg-qual 24 –output-vcf output.vcf

Variant annotations with Variant Effect Predictor (VEP) :
perl variant_effect_predictor.pl -i output.vcf --database --assembly GRCh37 --port 3337 --hgvs --force_overwrite --gmaf --refseq --numbers --symbol --field Uploaded_variation,Location,Allele,Feature,Consequence,cDNA_position,CDS_position,Protein_position,Amino_acids,Codons,Existing_variation,IMPACT,SYMBOL,EXON,HGVSc,HGVSp,GMAF,CLIN_SIG -o output.txt

Ion Reporter variant caller parameters

Analysis

Data Quality Stringency

Filter: Phred-scaled minimum average evidence per read or no-call. Related VCF field: MLLD. Allowed values: Decimal numbers >= 0. Recommended values >= 6.5

0 <= 10

Downsample To Coverage

Reduce coverage in over-sampled locations to this value. Allowed values: Integers >= 1.

1 <= 2000

Snp Min Cov Each Strand

Filter: Minimum coverage required on each strand. Allowed values: Integers >= 0. Recommended values >= 3.

0 <= 4

Snp Min Variant Score

Filter: Phred-scaled evidence that the reads support a variant above minimum frequency. Quality: Integers >= 0. Recommended values >= 10

0 <= 6

Snp Min Allele Freq

Frequency cutoff for supporting a variant. Allowed values: Decimal numbers between 0 and 1.0. Suggested trial value (TagSeq) between 0.0005 and 0.005, (other) between 0.01 and 0.2

0 <= 0.02 <= **1**

Snp Min Coverage

total coverage required of reads or no-call. Allowed values: Integers >= 0. Suggested trial value (TagSeq) between 2 and 10000, (other) between 5 and 20.

0 <= 100

SNP Strand Bias

Filter: proportion of variant alleles comes overwhelmingly from one strand. Related VCF field: STB. Allowed values: Decimal numbers between 0.5 and 1.0. Recommended 0.95

0.5 <=0.95 <= **1**

Indel Min Cov Each Strand

Filter: Minimum coverage required on each strand. Allowed values: Integers >= 0. Recommended >= 3

0 <= 4

Indel Min Variant Score

Filter: Phred-scaled evidence that the reads support a variant above minimum frequency. Quality: Integers >= 0. Recommended values >= 10.

0 <= 6

Indel Min Allele Freq

Frequency cutoff for supporting a variant. Allowed values: Decimal numbers between 0 and 1.0. Suggested trial value (TagSeq) between 0.0005 and 0.005, (other) between 0.05 and 0.2

0 <= 0.02 <= **1**

Indel Min Coverage

Total coverage required of reads or no-call. Allowed values: Integers >= 0. Suggested trial value (TagSeq) between 2 and 10000, (other) between 15 and 30.

0 <= 100

Indel Strand Bias

Filter: proportion of variant alleles comes overwhelmingly from one strand. Related VCF field: STB. Allowed values: Decimal numbers between 0.5 and 1.0. Recommended 0.95

0.5 <=0.9 <= **1**

Hotspot Min Cov Each Strand

Filter: Minimum coverage required on each strand. Allowed values: Integers >= 0. Recommended >= 3

0 <= 4

Hotspot Min Variant Score

Phred-scaled evidence that the reads support a variant above minimum frequency. Quality: Integers >= 0. Recommended values >= 10.

0 <= 6

Hotspot Min Allele Freq

Frequency cutoff for supporting a variant. Allowed values: Decimal numbers between 0 and 1.0. Suggested trial value (TagSeq) between 0.0005 and 0.005, (other) between 0.01 and 0.2.

0 <=0.01 <= **1**

Hotspot Min Coverage

Total coverage required of reads or no-call. Allowed values: Integers >=0. Suggested trial value (TagSeq) between 2 and 10000, (other) between 5 and 20.

0 <= 100

Hotspot Strand Bias

Filter: proportion of variant alleles comes overwhelmingly from one strand. Related VCF field: STB. Allowed values: Decimal numbers between 0.5 and 1.0. Recommended 0.95

0.5 <= 0.95 <= **1**

Prediction Precision

Number of pseudo-data-points suggesting our predictions match the measurements without bias. Allowed values: Decimal numbers >= 0 . Recommended value 1.0

0 <= 1.0

Outlier Probability

Prior probability that a read comes from some other distribution. Allowed values: Decimal numbers between 0 and 1.0. Recommended values between 0.005 and 0.01

0 <= 0.005 <= **1**

Heavy Tailed

How heavy the t-distribution tails are to allow for unusual spread in the data. Allowed values: Decimal numbers >= 0. Recommended value 3.0

0 <= 3

Filter Unusual Predictions

Filter: predictions are distorted to fit the data more than this distance (relative to the size of the variant). Related VCF fields: FWDB, REVB [RBI = sqrt(FWDB ^ 2 + REVB ^ 2)]. Allowed values: Decimal numbers >= 0. Recommended value: 0.3 = 30% of variant change size

0 <= 0.12

Filter Insertion Predictions

Filter: observed clusters deviate from predictions more than this amount (relative to the size of the variant). Related VCF fields: VARB, REFB. Allowed values: Decimal numbers >= 0. Recommended value: 0.2 = 20% of variant change size

0 <= 0.2

Filter Deletion Predictions

Filter: observed clusters deviate from predictions more than this amount (relative to the size of the variant). Related VCF fields: VARB, REFB. Allowed values: Decimal numbers >= 0. Recommended value: 0.2 = 20% of variant change size

0 <= 8

HP Max Length

Filter: homopolymer length involved in an in/del. Related VCF field: HRUN. Allowed values: Integers >= 1. Recommended value: 8

1 <= 8

Do SNP Realignment

Realign reads in the vicinity of SNP candidates.Allowed values: 0 = do not realign, 1 = realign. Recommended value: PGM: (germline) 0, (somatic) 1; Proton: (germline) 1, (somatic) 1

True **False**

Suppress Recalibration

Recalibration values from pipeline used or not (experimental). No related fields, changes basecalling behavior. Allowed values: 0 = allow recalibration, 1 = don't allow recalibration.

True **False**

SSE Probability Threshold

Filter out variants in motifs with error rates above this.

0 <= 1.0 <= **1**

Mnp Min Cov Each Strand

Filter: Minimum coverage required on each strand. Allowed values: Integers >= 0. Recommended values >= 3.

0 <= 4

Mnp Min Variant Score

Filter out MNPs with a QUAL score less than or equal to this Phred-scaled value.

0 <= 6

Mnp Min Allele Freq

Frequency cutoff for supporting a variant. Allowed values: Decimal numbers between 0 and 1.0. Suggested trial value (TagSeq) between 0.0005 and 0.005, (other) between 0.01 and 0.2

0 <= 0.02 <= **1**

Mnp Min Coverage

total coverage required of reads or no-call. Allowed values: Integers >= 0. Suggested trial value (TagSeq) between 2 and 10000, (other) between 5 and 20.

0 <= 100

MNP Strand Bias

Filter: proportion of variant alleles comes overwhelmingly from one strand. Related VCF field: STB. Allowed values: Decimal numbers between 0.5 and 1.0. Recommended 0.95

0.5 <=0.95 <= **1**

MNP Strand Bias Pval

filter out mnps with pval below this [1.0] given strand bias > mnp-strand-bias.

0 <= <= **1**

SNP Strand Bias Pval

filter out snps with pval below this [1.0] given strand bias > snp-strand-bias.

0 <= 0.01<= **1**

INDEL Strand Bias Pval

Filter out indels with pval below this [1.0] given strand bias > indel-strand-bias.

0 <= 1.0 <= **1**

Hotspot Strand Bias Pval

Filter out hotspot variants with pval below this [1.0] given strand bias > hotspot-strand-bias.

0 <=0.01 <= **1**

Position Bias Reference Fraction

Skip position bias filter if (reference read count)/(reference + alt allele read count) less than or equal to this.

0 <= 0.05 <= **1**

Position Bias

Filter out variants with position bias relative to soft clip ends in reads > position-bias.

0 <= 0.75<= **1**

Position Bias Pvalue

Filter out if position bias is above the Position Bias given pval less than Position Bias Pval.

0 <= 0.05<= **1**

Use position bias.

Enable the position bias filter.

True **False**

Indel As HPindel

Apply indel filters to non HP indels.

True **False**

Do MNP Realignment

Realign reads in the vicinity of candidate mnp variants.

True **False**

Realignment Threshold

Maximum allowed fraction of reads where realignment causes an alignment change.

0 <=0 <= **1**

FD Nonsnp Min Var Cov

Override min_var_coverage of the flow-disrupted variants that are not SNPs (0 to disable the override). Suggested trial value between 0 and 10. Impact: Decreasing values make variant calls less specific but more sensitive

0 <= 1<= **10**

Read Mismatch Limit

Do not use reads with number of mismatches (where 1 gap open counts 1) above this value. Allowed value: Integers >=0 where 0 to disable this filter. Suggested trial value (TagSeq) 5, (other) 0

0 <= 0

Min Cov Fraction

Do not use reads with fraction of covering the best assigned unmerged target region below this. Allowed values: Decimal numbers between 0 and 1. Suggested trial value (TagSeq) 0.9, (other) 0

0 <= 0

Use Input Allele Only

Only consider provided alleles in the hotspots file. Allowed values: 0 = generate de novo candidates, 1 = hotspots only

0 <= 0<= **1**

Liquid Biopsy

Min Family Size

Minimum number of reads with same UID required to form a functional family. Suggested value between 3 and 7. Impact: Increasing values make variant calls less sensitive but more specific.

0 < 3

SNP Min Var Coverage

Minimum number of variant supporting functional families required to make a SNP call. Suggested trial value between 2 and 10. Impact: Increasing values make variant calls less sensitive but more specific

2 <= 2<= **10**

MNP Min Var Coverage

Minimum number of variant supporting functional families required to make a MNP call. Suggested trial value between 2 and 10. Impact: Increasing values make variant calls less sensitive but more specific

2 <= 2 <= **10**

INDEL Min Var Coverage

Minimum number of variant supporting functional families required to make a INDEL call. Suggested trial value between 2 and 10. Impact: Increasing values make variant calls less sensitive but more specific

2 <= 2R<= **10**

Hotspot Min Var Coverage

Minimum number of variant supporting functional families required to make a hotspot call. Suggested trial value between 2 and 10. Impact: Increasing values make variant calls less sensitive but more specific

2 <=2 <= **10**

Indel Func Size Offset

require family of size >= (min_tag_fam_size + this value) to be functional for calling HP-INDEL. Suggested trial value between 0 and 4. Impact: Increasing values make variant calls less sensitive but more specific

0 <=2 <= **4**

Tag Sim Max Cov

Check the similarity of UID of variant families if the variant molecular coverage is less than or equal to this value. Related VCF field: TGSM. Suggested trial value 20

0 <= 20
